# The association between gambling frequency and risk of harm: Analysis using health survey data from England and Scotland

**DOI:** 10.1111/add.70344

**Published:** 2026-02-17

**Authors:** Esther Moore, Robert Pryce, Hazel Squires, Elizabeth Goyder

**Affiliations:** ^1^ Sheffield Centre for Health and Related Research (SCHARR) University of Sheffield Sheffield UK

**Keywords:** gambling, gambling frequency, gambling‐related harm, Problem Gambling Severity Index, public health, risk of harm

## Abstract

**Background and aims:**

Health economic models can be used to assess the effectiveness and cost‐effectiveness of public health policies for gambling. To develop such a model, we must understand how gambling behaviour is associated with risk of experiencing gambling‐related harms. This study aimed to: (1) assess the strength of association between gambling frequency and the risk of gambling‐related harm and to examine how these associations differ when lottery‐only players are excluded; (2) apply the study's findings in a hypothetical policy model aimed at reducing gambling frequency.

**Design:**

Observational study using six waves of cross‐sectional data from the Health Survey for England and the Scottish Health Survey.

**Setting:**

Survey conducted in England in 2015, 2016 and 2018 and Scotland in 2015, 2016 and 2017.

**Participants:**

The sample included 16 648 adults (aged 18 and over) who reported gambling in the past year, generally representative of the populations of England and Scotland.

**Measurements:**

Gambling frequency was measured using 6 categories which indicated frequency in the past 12 months: (a) 2 or more times a week; (b) once a week; (c) less than once a week, more than once a month; (d) once a month; (e) every 2–3 months; (f) once or twice a year. Risk of gambling‐related harm was assessed using Problem Gambling Severity Index (PGSI) score (0–27) and its four categories: no‐risk (0), low‐risk (1–2), moderate‐risk (3–7) and high‐risk (≥8). Control variables included age, sex, deprivation, social grade, presence of mental disorder and frequency of drinking alcohol.

**Findings:**

Using multinomial logistic regression and zero‐inflated negative binomial models we found that gambling at least twice weekly was associated with a statistically significantly higher PGSI score than gambling once or twice a year (incidence rate ratio = 3.528, 95% confidence interval = 2.040–6.103, *P* value < 0.001). Reducing gambling to guideline levels for people gambling at least twice weekly moved 10% of the sub‐sample from higher PGSI categories (low, medium and high risk) to the no‐risk category and shifted the distribution of PGSI scores down.

**Conclusions:**

There appears to be a statistically significant association between gambling frequency and risk of gambling‐related harm. Data derived from this and similar analyses can be used to model gambling policies which impact gambling frequency.

## INTRODUCTION

Gambling‐related harms are a public health issue estimated to cost the UK government £412.9 million each year in direct expenses such as healthcare, crime and unemployment services [1]. When wider societal impacts are included, such as reduced quality of life resulting from depression, the total cost rises to approximately £1.05–£1.77 billion annually [1]. Consequently, there have been calls to introduce tighter regulations of the gambling industry in the UK to reduce gambling‐related harm [2, 3]. While a 2023 review of the Gambling Act 2005 outlined potential regulatory changes, it also highlighted the lack of evidence for some policies [4]. As many gambling policies aim to reduce harm by changing gambling behaviour, understanding the association between gambling behaviour and risk of harm is essential for maximising policy effectiveness and developing health economic models.

Health economic models can help us to understand the potential impact, costs and benefits of proposed interventions, or the wider/longer term impact of interventions that have already been implemented [5]. Public health economic models have not yet been used in gambling, and this could be partly because of the lack of good epidemiological evidence on the association between gambling behaviour and risk of harm. Areas where public health economic models are well established, such as for alcohol and tobacco, have strong epidemiological evidence about how the consumption of these products impacts the risk of harm [6, 7]. However, evidence on the relationship between gambling consumption/behaviour and risk of harm is complex because of a lack of standardised measurement methods. Gambling behaviour can be conceptualised in a number of ways, including frequency, duration, spending and the breadth of products used [8]. Harm can also be measured using a number of approaches, including specific gambling‐related harm measures [9, 10], problem gambling measures used as proxies [11] and specific harm indicators (e.g. depression attributable to gambling) [12]. The complexity of the relationship between gambling behaviour and harms is compounded by evidence suggesting gambling‐related harm can vary across activities, with certain product types exhibiting a higher concentration of spending [13].A recent systematic review examined the evidence between gambling consumption and gambling‐related harm [14]. The review found studies most often used gambling expenditure as the measure of consumption, followed by frequency and the percentage of income spent on gambling. Harm was primarily measured using the Problem Gambling Severity Index (PGSI), followed by the South Oaks Gambling Screen (SOGS) and Diagnostic and Statistical Manual of Mental Disorders (DSM)‐related measures. These measures were not designed to measure gambling‐related harm, with many items in these instruments indicating behavioural addiction rather than the negative consequences of gambling [15]. It would be more appropriate to consider them as indictors of risk of harm rather than a measure of harm itself. Seven out of 17 studies in the review visually described the relationship between gambling consumption and harm by plotting risk curves. The remaining 10 studies examined either the concentration of gambling among those experiencing moderate risk/problem gambling or explored the applicability of the total consumption model to gambling. While these articles offer insights into the relationship between gambling consumption and gambling‐related harm, they do not quantify this association (e.g. using relative risk ratios) in a manner suitable for predicting policy outcomes within health economic models.

Data from the UK on gambling behaviour and risk of harm are limited. The British Gambling Prevalence Survey (BGPS) ran in 1999, 2007 and 2010, and included questions on gambling frequency and gambling expenditure [16]. All waves measured problem gambling using DSM‐IV; the 1999 wave also included SOGS and the 2007/2010 waves used PGSI. These data are now at least 15 years old and do not reflect recent changes in the gambling industry, notably the Gambling Act 2005 and the increase in online gambling. While net expenditure on gambling theoretically provides a more accurate estimate of risk of harm, its measurement is challenging because of the likely under‐reporting of losses (e.g. ‘losses disguised as wins’) [17]. More recent surveys in the UK have only asked about frequency rather than expenditure. These include the Health Survey for England (HSE) and the Scottish Health Survey (SHeS), which included questions on both gambling frequency and the PGSI in waves from 2015 to 2018 [18, 19]. Owing to the issues with measuring expenditure and the age of the BGPS, this study used the more recent data from HSE and SHeS. Given the cross‐sectional nature of these data, we investigated the observational association between gambling frequency and the risk of gambling‐related harm, as indicated by the PGSI.

The study had two aims: (i) assess the strength of association between gambling frequency and the risk of gambling‐related harm, and examine how these associations differ when lottery‐only players are excluded; and (ii) apply the study findings in a hypothetical policy model aimed at reducing gambling frequency.

## METHODS

A protocol for the analysis was pre‐registered on the Open Science Framework (OSF) [20]. The protocol included two transformations of the categorical gambling frequency measure into a continuous variable. Both methods relied on unsupported assumptions and did not add meaningful insight, so this part of the analysis was removed during peer review. The results are available on the OSF, for transparency [20]. Any other deviations from the protocol are specified in the relevant section below.

### Design

Three waves of the HSE, in 2015, 2016 and 2018, and three waves of the SHeS, in 2015, 2016 and 2017, were combined into one data set. Both the HSE and the SHeS are large cross‐sectional surveys that have approximately 8000 and 5000 adults, respectively, each year and cover various health‐related topics [18, 19]. Data are collected via face‐to‐face in‐home interviews and self‐completion questionnaire. The gambling questions are part of the self‐completion questions in both surveys. In the HSE a random subsample of adults aged 16 years and over are asked the gambling questions; in the SHeS all adults are asked the gambling questions. The same questions related to gambling were asked, using the same wording, in both the HSE and the SHeS. These included: the nine‐item PGSI; the question ‘Have you spent any money on any of the following activities in the last 12 months?’, which was followed by a list of gambling activities including lotteries; and ‘Thinking about all the activities covered in the previous question, would you say you spend money on these activities: 2 or more times a week; once a week; less than once a week, more than once a month; once a month; every 2–3 months; or once or twice a year?’ These questions were also included in the 2021 wave; however, this was excluded because of changes in data collection during the COVID‐19 pandemic [21].

### Sample

**FIGURE 1:**
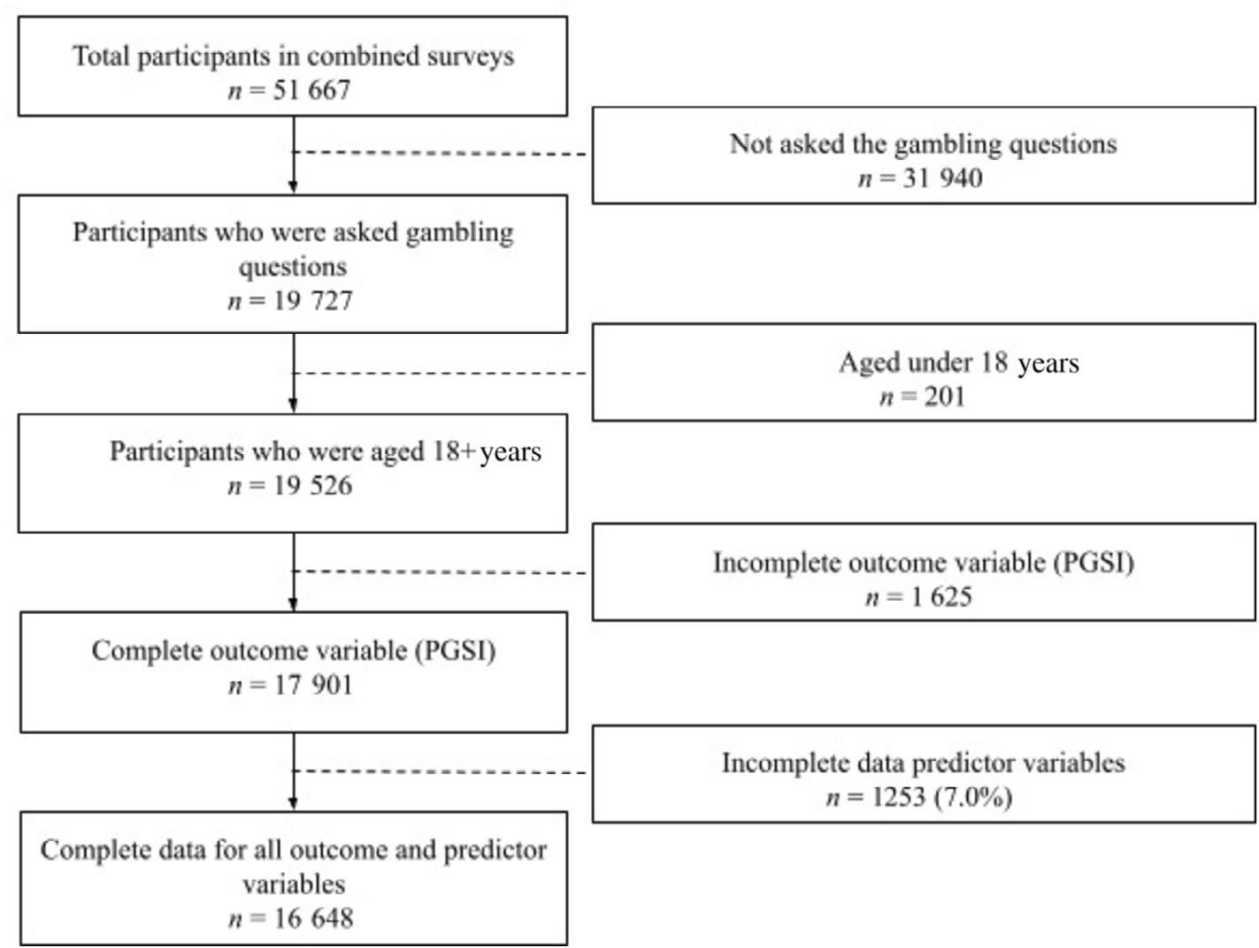
A flow diagram showing the selection of the final analytic sample. PGSI = problem gambling severity index.

The analytic sample was restricted based on the following inclusion criteria: participants had to be aged 18 years or over, have been asked the gambling questions, have a complete PGSI score (the dependent variable) and have no missing data on any variables included in the analysis. Participants who did not meet these criteria were excluded, resulting in a final analytic sample of 16 648 (Figure 1).

### Measures

#### Outcomes

We had two outcomes of interest: the PGSI score and the PGSI category. The PGSI category is derived from the PGSI score (ranging from 0 to 27) in the following way: no‐risk category if PGSI score = 0; low‐risk category if PGSI = 1–2; moderate‐risk category if PGSI = 3–7; and high‐risk category if PGSI score is ≥8 [22].

#### Exposures

Participants were asked the following question in relation to their gambling frequency: ‘Thinking about all the activities covered in the previous question, would you say you spend money on these activities: 2 or more times a week; once a week; less than once a week, more than once a month; once a month; every 2–3 months; or once or twice a year?’

#### Control variables

Identifying and controlling for potential independent risk factors for both gambling participation and problem gambling is essential for isolating the true association between gambling frequency and risk of gambling‐related harm. A 2023 review by Public Health England [23], using a socio‐ecological approach, examined potential risk factors for gambling participation and harmful gambling (defined as being in the low, moderate or high PGSI category). Although most studies were of low quality, some evidence suggested relationships between gender [24, 25, 26, 27, 28, 29], accessibility [26, 28, 30, 31, 32, 33] and density of gambling outlets [31], proximity to gambling outlets [31], certain mental health conditions (including depression) [24, 27, 29, 33, 34, 35], deprivation [36], socio‐economic status [27, 37] and substance use (including alcohol consumption) [24, 33, 35, 38, 39] with both gambling participation and problem gambling. While this review found no conclusive evidence on age, a more recent analysis indicated that younger adults were significantly more likely to experience problem gambling than middle‐aged adults, who in turn were more likely to experience problem gambling than older adults [40]. Furthermore, UK data show age‐related variations in gambling participation [41]. Based on this evidence, age, sex, level of deprivation, social grade, presence of mental disorder and frequency of alcohol consumption were included as control variables in our models. Table 1 shows a description of each control variable. In a deviation from the protocol [20], age was recoded into four categories: 18–34 years; 35–49 years; 50–64 years; and 65+ years. This was done to ensure an adequate sample size in each age group and PGSI category, which allowed for model convergence.

**TABLE 1:** A description of the control variables included in the models.

Variable	Description
Age	In the HSE, age is grouped into 3‐year age bands. In the SHeS, age is grouped in single years. For this analysis, age was recoded into four categories: 18–34 years; 35–49 years; 50–64 years; and 65+ years. This was done to ensure an adequate sample size in each age group and PGSI category.
Sex	Sex was included as a binary variable, either female or male.
Level of deprivation	Level of deprivation is measured independently in England and Scotland. In England the Index of Multiple Deprivation [42] is used and in Scotland the Scottish Index of Multiple Deprivation [43] is used. For this analysis, we created a new variable called deprivation, which had 10 categories: Scotland—1 (least deprived), Scotland—2, Scotland—3, Scotland—4, Scotland—5 (most deprived), England—1 (least deprived), England—2, England—3, England—4 and England—5 (most deprived).
Social grade	Participants were asked about their job using the NS‐SEC [44]. This is a measure with eight levels: (i) higher managerial and professional occupations; (ii) lower managerial and professional occupations; (iii) intermediate occupations; (iv) small employers and own account workers; (v) lower supervisory and technical occupations; (vi) semi‐routine occupations; (vii) routine occupations; and (viii) never worked and long‐term unemployed. The HSE and SHeS both included an additional ‘other’ category.
Presence of mental disorder	Both surveys included a binary variable that indicates whether the participant has a diagnosed mental health disorder.
Frequency of drinking alcohol	Participants were asked how often they drank alcohol, with the following eight categories: (i) almost every day; (ii) five or six days a week; (iii) three or four days a week; (iv) once or twice a week; (v) once or twice a month; (vi) once every couple of months; (vii) once or twice a year; and (viii) not at all in the last 12 months/non‐drinker.

Abbreviations: HSE = Health Survey for England; NS‐SEC = National Statistics Socio‐economic Classification; PGSI = Problem Gambling Severity Index; SHeS = Scottish Health Survey.

### Analyses

All analyses were carried out using STATA 18 and the code is available from GitHub at: https://github.com/esther-moore/gambling-freq-harm-risk.

#### Descriptive statistics

Descriptive statistics were used to summarise the sample characteristics across PGSI categories. Frequencies and proportions were used for categorical variables, while the mean and standard error were presented for PGSI score, the only continuous variable. Differences across PGSI categories were explored using chi‐square tests; the PGSI score was not compared across PGSI categories, as the categories are directly derived from the score itself.

#### The association between gambling frequency and PGSI category (full model)

A multinomial logistic model was used to estimate the probability of being in the low‐risk, moderate‐risk and high‐risk PGSI categories based on gambling frequency. This is a deviation from the published protocol, where it specified that an ordered logistic regression would be used [20]. The proportional odds assumption was not met, so a multinomial logistic model was used as a suitable alternative [45]. In this model, the PGSI no‐risk category was used as the reference group. This means each estimate was the probability of being in the specified PGSI category, compared with being in the no‐risk category. We controlled for the demographic/socio‐economic factors outlined in the above section. We calculated relative risk ratios (RRRs) from the multinomial logistic regression by exponentiating the estimated coefficients and plotted the predicted probabilities.

#### The association between gambling frequency and PGSI score (full model)

A zero‐inflated negative binomial was used to predict the PGSI score based upon gambling frequency. We also ran a negative binomial model, but the zero‐inflated model showed a better fit when considering the Akaike information criterion (AIC) and the Bayesian information criterion (BIC) (Table S1). The same control variables were included as used in the analysis of PGSI category. We calculated incidence rate ratios (IRRs) from the count component of the zero‐inflated negative binomial regression model by exponentiating the estimated coefficients.

#### The association controlling only for age, sex and deprivation (reduced model)

In a deviation from the protocol, we ran the same models controlling only for age, sex and Index of Multiple Deprivation (IMD) quintile to improve the useability and generalisability of our findings. These variables were selected as they are commonly available in most surveys. Age and sex are routinely collected, and it is possible to derive the IMD quintile from a participant's postcode. In contrast, the National Statistics Socio‐economic Classification (NS‐SEC) category, mental health status and frequency of drinking alcohol are less likely to be included or may be measured differently than in the HSE and SHeS. Only including age, sex and IMD therefore increases the applicability of our results to external data sets and health economic models.

### Sensitivity analysis—excluding people who only gamble on lotteries

Previous work has suggested that people who only gamble on the lottery might distort the association between gambling frequency and harm because this population might gamble relatively frequently (i.e. once weekly) but are doing so on what could be considered a lower risk gambling activity [46]. Although there is only one question on frequency related to the frequency of gambling on any forms, the HSE and SHeS do include binary (yes/no) questions related to participation in different forms of gambling. It was possible to exclude people who report only gambling on lotteries (i.e. they have responded ‘yes’ to the questions related to participation of lotteries but ‘no’ to all other forms of gambling) as a sensitivity analysis. We therefore repeated the analyses using the full model (controlling for age, sex, level of deprivation, social grade, presence of mental disorder and frequency of drinking alcohol) but excluded anyone in the sample who reported only gambling on a type of lottery, reducing the sample size to 9320. We qualitatively compared the 95% confidence intervals (95% CIs) of the RRRs and IRRs from this analysis and the main model to assess potential differences.

### Hypothetical modelling of a policy to reduce gambling frequency

To look at a hypothetical policy that reduces gambling frequency to be in line with the lower risk gambling guidelines we took a subsample of the initial sample who gambled above the lower risk guideline levels. We first used the multinomial logit model and estimated the percentage of the affected population that would be in each PGSI category using the baseline gambling frequency. We then reduced the gambling frequency to be in line with the guidelines. The Canadian lower risk guidelines for frequency are to gamble no more than four times per month (i.e. 48 days per year) [47] and the Australian lower risk guidelines are to gamble no more than once per week (i.e. 52 days per year) [48]. Anyone in the ‘2 or more times a week’ category was moved into the ‘Once a week’ category. Using the same model, we predicted the percentage of the population that would be in each PGSI category again and looked at the difference in the distribution. For PGSI score we used a similar approach but used the zero‐inflated negative binomial model to predict the PGSI score before and after gambling frequency was reduced.

## RESULTS

### Descriptive statistics

Table 2 displays a description of the sample by PGSI category.

**TABLE 2:** The descriptive statistics of the analytic sample.

	PGSI category					
	No risk	Low risk	Moderate risk	High risk	Total	χ^2^ test
	*n* (%)	*n* (%)	*n* (%)	*n* (%)	*n* (%)	
Total	15 673 (94.1%)	619 (3.7%)	239 (1.4%)	117 (0.7%)	16 648 (100.0%)	
Sex						
Male	7197 (45.9%)	459 (74.2%)	186 (77.8%)	100 (85.5%)	7942 (47.7%)	<0.001
Female	8476 (54.1%)	160 (25.8%)	53 (22.2%)	17 (14.5%)	8706 (52.3%)	
Age						
18–34 years	3172 (20.2%)	285 (46.0%)	112 (46.9%)	48 (41.0%)	3617 (21.7%)	<0.001
35–49 years	4251 (27.1%)	170 (27.5%)	66 (27.6%)	36 (30.8%)	4523 (27.2%)	
50–64 years	4543 (29.0%)	116 (18.7%)	46 (19.2%)	27 (23.1%)	4732 (28.4%)	
65+ years	3707 (23.7%)	48 (7.8%)	15 (6.3%)	6 (5.1%)	3776 (22.7%)	
Index of Multiple Deprivation for England and Scotland						
1—England (least deprived, ref.)	2138 (13.6%)	77 (12.4%)	17 (7.1%)	6 (5.1%)	2238 (13.4%)	<0.001
1—Scotland (least deprived)	1081 (6.9%)	30 (4.8%)	8 (3.3%)	2 (1.7%)	1121 (6.7%)	
2—England	2213 (14.1%)	88 (14.2%)	21 (8.8%)	11 (9.4%)	2333 (14.0%)	
2—Scotland	1257 (8.0%)	39 (6.3%)	12 (5.0%)	11 (9.4%)	1319 (7.9%)	
3—England	2281 (14.6%)	75 (12.1%)	39 (16.3%)	17 (14.5%)	2412 (14.5%)	
3—Scotland	1104 (7.0%)	29 (4.7%)	14 (5.9%)	7 (6.0%)	1154 (6.9%)	
4—England	2017 (12.9%)	94 (15.2%)	33 (13.8%)	18 (15.4%)	2162 (13.0%)	
4—Scotland	970 (6.2%)	35 (5.7%)	11 (4.6%)	4 (3.4%)	1020 (6.1%)	
5—England (most deprived)	1797 (11.5%)	111 (17.9%)	58 (24.3%)	28 (23.9%)	1994 (12.0%)	
5—Scotland (most deprived)	815 (5.2%)	41 (6.6%)	26 (10.9%)	13 (11.1%)	895 (5.4%)	
NS‐SEC (occupation)						
Higher managerial and professional occupations	1938 (12.4%)	60 (9.7%)	17 (7.1%)	2 (1.7%)	2017 (12.1%)	<0.001
Lower managerial and professional occupations	4012 (25.6%)	142 (22.9%)	52 (21.8%)	27 (23.1%)	4233 (25.4%)	
Intermediate occupations	2364 (15.1%)	77 (12.4%)	22 (9.2%)	9 (7.7%)	2472 (14.8%)	
Small employers and own account workers	1397 (8.9%)	59 (9.5%)	27 (11.3%)	11 (9.4%)	1494 (9.0%)	
Lower supervisory and technical occupations	1181 (7.5%)	55 (8.9%)	25 (10.5%)	8 (6.8%)	1269 (7.6%)	
Semi‐routine occupations	2765 (17.6%)	116 (18.7%)	46 (19.2%)	22 (18.8%)	2949 (17.7%)	
Routine occupations	1909 (12.2%)	93 (15.0%)	46 (19.2%)	34 (29.1%)	2082 (12.5%)	
Never worked and long‐term unemployed	67 (0.4%)	6 (1.0%)	0 (0.0%)	4 (3.4%)	77 (0.5%)	
Other	40 (0.3%)	11 (1.8%)	4 (1.7%)	0 (0.0%)	55 (0.3%)	
Long‐term mental health disorder						
No	14 626 (93.3%)	557 (90.0%)	213 (89.1%)	93 (79.5%)	15 489 (93.0%)	<0.001
Yes	1047 (6.7%)	62 (10.0%)	26 (10.9%)	24 (20.5%)	1159 (7.0%)	
Frequency drunk alcohol in past 12 months						
Almost every day	1298 (8.3%)	45 (7.3%)	23 (9.6%)	13 (11.1%)	1379 (8.3%)	0.424
Five or six days a week	689 (4.4%)	26 (4.2%)	10 (4.2%)	3 (2.6%)	728 (4.4%)	
Three or four days a week	2391 (15.3%)	114 (18.4%)	42 (17.6%)	13 (11.1%)	2560 (15.4%)	
Once or twice a week	5065 (32.3%)	211 (34.1%)	85 (35.6%)	39 (33.3%)	5400 (32.4%)	
Once or twice a month	2509 (16.0%)	93 (15.0%)	32 (13.4%)	20 (17.1%)	2654 (15.9%)	
Once every couple of months	1340 (8.5%)	58 (9.4%)	17 (7.1%)	11 (9.4%)	1426 (8.6%)	
Once or twice a year	1279 (8.2%)	42 (6.8%)	15 (6.3%)	7 (6.0%)	1343 (8.1%)	
Not at all in the last 12 months/non‐drinker	1102 (7.0%)	30 (4.8%)	15 (6.3%)	11 (9.4%)	1158 (7.0%)	
Frequency of spending money on any gambling activities						
2 or more times a week	1849 (11.8%)	190 (30.7%)	119 (49.8%)	64 (54.7%)	2222 (13.3%)	<0.001
Once a week	4538 (29.0%)	162 (26.2%)	62 (25.9%)	29 (24.8%)	4791 (28.8%)	
Less than once a week, more than once a month	1512 (9.6%)	108 (17.4%)	21 (8.8%)	9 (7.7%)	1650 (9.9%)	
Once a month	1830 (11.7%)	67 (10.8%)	15 (6.3%)	11 (9.4%)	1923 (11.6%)	
Every 2–3 months	2051 (13.1%)	55 (8.9%)	13 (5.4%)	3 (2.6%)	2122 (12.7%)	
Once or twice a year	3893 (24.8%)	37 (6.0%)	9 (3.8%)	1 (0.9%)	3940 (23.7%)	
Mean PGSI score (standard error)	0.000 (0.000)	1.263 (0.441)	4.335 (1.346)	13.453 (5.686)	0.204 (1.348)	NA

Abbreviations: NS‐SEC = National Statistics Socio‐economic classification; PGSI = Problem Gambling Severity Index.

### The association between gambling frequency and PGSI category (full model)

**FIGURE 2:**
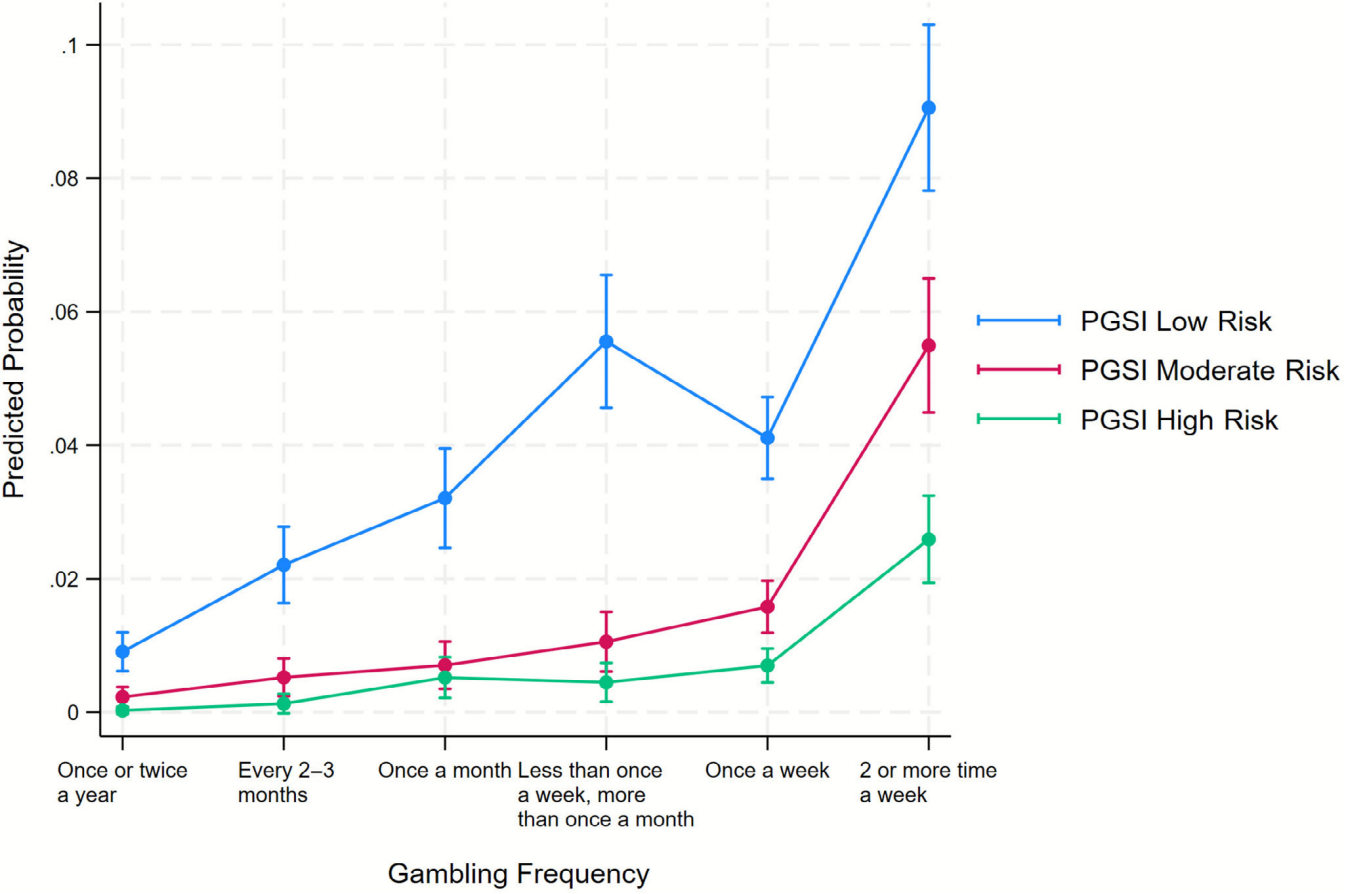
Predicted probabilities of PGSI categories across levels of gambling frequency. PGSI = problem gambling severity index.

Table 3 displays the results of the multinomial logistic regression. The results show a significant association between gambling frequency and the likelihood of being in the higher risk PGSI categories. Our analysis used individuals who gambled only once or twice a year as the reference group. Compared with this group, individuals who gambled at least twice a week showed significantly increased odds across the risk categories: they were 15 times more likely to be in the low‐risk category compared with the no‐risk category (model 1); 38 times more likely to be in the moderate‐risk category compared with the no‐risk category; and 153 times more likely to be in the high‐risk category compared with the no‐risk category. The association between gambling every 2–3 months and the RRR of being in the high‐risk category was the only statistically insignificant result. This is likely to be associated with the very small sample size, with only three individuals gambling at this frequency and being in the high‐risk category (Table 2). Figure 2 shows the predicted probability of individuals falling into different PGSI risk categories (low risk, moderate risk and high risk) as a function of their reported gambling frequency. There is a general increase in the probability of being in each of the low‐, moderate‐ and high‐risk categories as gambling frequency increases.

**TABLE 3:** The results of the multinomial logistic regression looking at the association between PGSI category and gambling frequency.

	Model 1		
	RRR	95% CI	*P*
**PGSI category: no risk**	Reference group		
**PGSI category: low risk**			
Frequency of spending money on gambling activities in the past 12 months			
Once or twice a year (ref.)	1	(1, 1)	
Every 2–3 months	2.541	(1.661, 3.887)	0.000
Once a month	3.847	(2.549, 5.804)	(0.000)
Less than once a week, more than once a month	7.106	(4.834, 10.44)	(0.000)
Once a week	5.180	(3.584, 7.486)	(0.000)
2 or more times a week	14.92	(10.29, 21.63)	(0.000)
Age			
18–34 years (ref.)	1	(1, 1)	(.)
35–49 years	0.389	(0.316, 0.479)	(0.000)
50–64 years	0.196	(0.155, 0.249)	(0.000)
65+ years	0.0891	(0.0641, 0.124)	(0.000)
Sex			
Male (ref.)	1	(1, 1)	(.)
Female	0.319	(0.261, 0.389)	(0.000)
NS‐SEC (occupation)			
Higher managerial and professional occupations (ref.)	1	(1, 1)	(.)
Lower managerial and professional occupations	1.255	(0.913, 1.725)	(0.163)
Intermediate occupations	1.431	(0.996, 2.056)	(0.053)
Small employers and own account workers	1.415	(0.966, 2.073)	(0.075)
Lower supervisory and technical occupations	1.085	(0.734, 1.604)	(0.681)
Semi‐routine occupations	1.536	(1.091, 2.162)	(0.014)
Routine occupations	1.331	(0.934, 1.896)	(0.113)
Never worked and long‐term unemployed	1.645	(0.630, 4.295)	(0.309)
Other	4.415	(2.008, 9.711)	(0.000)
Frequency of alcohol intake in the past 12 months			
Almost every day (ref.)	1	(1, 1)	(.)
Five or six days a week	1.101	(0.659, 1.839)	(0.714)
Three or four days a week	1.263	(0.872, 1.829)	(0.217)
Once or twice a week	1.008	(0.711, 1.428)	(0.965)
Once or twice a month	0.875	(0.594, 1.288)	(0.498)
Once every couple of months	1.162	(0.762, 1.772)	(0.486)
Once or twice a year	1.040	(0.663, 1.632)	(0.865)
Not at all in the last 12 months/non‐drinker	0.765	(0.468, 1.252)	(0.286)
Long‐term mental health disorder			
No	1	(1, 1)	(.)
Yes	1.685	(1.264, 2.248)	(0.000)
Index of Multiple Deprivation for England and Scotland			
1—England (least deprived, ref.)	1	(1, 1)	(.)
1—Scotland (least deprived)	0.662	(0.425, 1.031)	(0.068)
2—England	1.049	(0.758, 1.452)	(0.773)
2—Scotland	0.700	(0.466, 1.052)	(0.086)
3—England	0.774	(0.552, 1.084)	(0.136)
3—Scotland	0.561	(0.358, 0.880)	(0.012)
4—England	0.999	(0.722, 1.382)	(0.995)
4—Scotland	0.686	(0.447, 1.052)	(0.084)
5—England (most deprived)	1.221	(0.886, 1.682)	(0.222)
5—Scotland (most deprived)	0.871	(0.575, 1.318)	(0.513)
**PGSI category: moderate risk**			
Frequency of spending money on gambling activities in the past 12 months			
Once or twice a year (ref.)	1	(1, 1)	(.)
Every 2–3 months	2.397	(1.019, 5.639)	(0.045)
Once a month	3.382	(1.470, 7.784)	(0.004)
Less than once a week, more than once a month	5.443	(2.472, 11.99)	(0.000)
Once a week	8.037	(3.955, 16.33)	(0.000)
2 or more times a week	37.85	(18.88, 75.88)	(0.000)
Age			
18–34 years (ref.)	1	(1, 1)	(.)
35–49 years	0.343	(0.247, 0.475)	(0.000)
50–64 years	0.151	(0.104, 0.219)	(0.000)
65+ years	0.0505	(0.0287, 0.0888)	(0.000)
Sex			
Male (ref.)	1	(1, 1)	(.)
Female	0.300	(0.215, 0.418)	(0.000)
NS‐SEC (occupation)			
Higher managerial and professional occupations (ref.)	1	(1, 1)	(.)
Lower managerial and professional occupations	1.449	(0.822, 2.552)	(0.200)
Intermediate occupations	1.312	(0.678, 2.541)	(0.420)
Small employers and own account workers	1.903	(1.009, 3.589)	(0.047)
Lower supervisory and technical occupations	1.354	(0.708, 2.587)	(0.359)
Semi‐routine occupations	1.664	(0.918, 3.016)	(0.093)
Routine occupations	1.639	(0.907, 2.962)	(0.102)
Never worked and long‐term unemployed	0.00000161	NE	(0.982)
Other	4.174	(1.204, 14.47)	(0.024)
Frequency of alcohol intake in the past 12 months			
Almost every day (ref.)	1	(1, 1)	(.)
Five or six days a week	1.047	(0.480, 2.286)	(0.908)
Three or four days a week	1.053	(0.610, 1.817)	(0.853)
Once or twice a week	0.904	(0.548, 1.491)	(0.692)
Once or twice a month	0.683	(0.382, 1.220)	(0.198)
Once every couple of months	0.781	(0.400, 1.524)	(0.468)
Once or twice a year	0.805	(0.403, 1.607)	(0.538)
Not at all in the last 12 months/non‐drinker	0.885	(0.443, 1.771)	(0.731)
Long‐term mental health disorder			
No (ref.)	1	(1, 1)	(.)
Yes	1.869	(1.204, 2.900)	(0.005)
Index of Multiple Deprivation for England and Scotland			
1—England (least deprived, ref.)	1	(1, 1)	(.)
1—Scotland (least deprived)	0.797	(0.337, 1.885)	(0.606)
2—England	1.147	(0.595, 2.211)	(0.683)
2—Scotland	0.913	(0.426, 1.955)	(0.814)
3—England	1.792	(0.994, 3.231)	(0.052)
3—Scotland	1.110	(0.533, 2.313)	(0.781)
4—England	1.498	(0.816, 2.751)	(0.192)
4—Scotland	0.904	(0.411, 1.989)	(0.802)
5—England (most deprived)	2.792	(1.577, 4.943)	(0.000)
5—Scotland (most deprived)	2.174	(1.131, 4.180)	(0.020)
**PGSI category: high risk**			
Frequency of spending money on gambling activities in the past 12 months			
Once or twice a year (ref.)	1	(1, 1)	(.)
Every 2–3 months	4.880	(0.506, 47.05)	(0.170)
Once a month	20.74	(2.667, 161.3)	(0.004)
Less than once a week, more than once a month	19.21	(2.424, 152.2)	(0.005)
Once a week	29.66	(4.019, 218.8)	(0.001)
2 or more times a week	153.4	(21.101, 115.7)	(0.000)
Age			
18–34 years (ref.)	1	(1, 1)	(.)
35–49 years	0.437	(0.275, 0.693)	(0.000)
50–64 years	0.205	(0.123, 0.341)	(0.000)
65+ years	0.0467	(0.0194, 0.112)	(0.000)
Sex			
Male (ref.)	1	(1, 1)	(.)
Female	0.144	(0.0838, 0.249)	(0.000)
NS‐SEC (occupation)			
Higher managerial and professional occupations (ref.)	1	(1, 1)	(.)
Lower managerial and professional occupations	6.662	(1.567, 28.33)	(0.010)
Intermediate occupations	5.148	(1.093, 24.24)	(0.038)
Small employers and own account workers	6.511	(1.420, 29.86)	(0.016)
Lower supervisory and technical occupations	3.560	(0.742, 17.07)	(0.112)
Semi‐routine occupations	6.999	(1.605, 30.52)	(0.010)
Routine occupations	9.815	(2.304, 41.80)	(0.002)
Never worked and long‐term unemployed	17.50	(2.663, 115.0)	(0.003)
Other	0.0000142	NE	(0.989)
Frequency of alcohol intake in the past 12 months			
Almost every day (ref.)	1	(1, 1)	(.)
Five or six days a week	0.604	(0.164, 2.220)	(0.448)
Three or four days a week	0.669	(0.297, 1.503)	(0.330)
Once or twice a week	0.863	(0.438, 1.700)	(0.671)
Once or twice a month	0.856	(0.401, 1.829)	(0.688)
Once every couple of months	1.062	(0.452, 2.493)	(0.891)
Once or twice a year	0.706	(0.267, 1.865)	(0.482)
Not at all in the last 12 months/non‐drinker	1.254	(0.531, 2.961)	(0.605)
Long‐term mental health disorder			
No (ref.)	1	(1, 1)	(.)
Yes	3.495	(2.107, 5.800)	(0.000)
Index of Multiple Deprivation for England and Scotland			
1—England (least deprived, ref.)	1	(1, 1)	(.)
1—Scotland (least deprived)	0.587	(0.116, 2.964)	(0.519)
2—England	1.719	(0.624, 4.737)	(0.295)
2—Scotland	2.311	(0.832, 6.424)	(0.108)
3—England	2.013	(0.777, 5.211)	(0.150)
3—Scotland	1.381	(0.450, 4.233)	(0.573)
4—England	1.897	(0.733, 4.908)	(0.187)
4—Scotland	0.731	(0.199, 2.679)	(0.636)
5—England (most deprived)	2.740	(1.095, 6.857)	(0.031)
5—Scotland (most deprived)	2.408	(0.877, 6.614)	(0.088)
Observations	16 648		
Pseudo *R* ^2^	0.189		

Abbreviations: PGSI = problem gambling severity index; NE = 95% confidence interval calculation not feasible owing to extremely small coefficient magnitude; NS‐SEC = National Statistics Socio‐economic Classification.

### The association between gambling frequency and PGSI category controlling only for age, sex and deprivation (reduced model)

While there were minor qualitative differences in the magnitude of the coefficients between model 1 and model 1a (the reduced model), the 95% CIs overlapped with those of the main analysis, suggesting no substantial differences in the estimated associations. The full results can be found in Table S2.

### The association between gambling frequency and PGSI score (full model)

Table 4 displays the results of the zero‐inflated negative binomial model, with the coefficients displayed as IRRs. Compared with gambling once or twice a year, only gambling once a week or at least twice a week was associated with a significantly higher PGSI score, with IRRs of 2.3 and 3.5, respectively.

**TABLE 4:** The results of the zero‐inflated negative binomial models looking at the association between PGSI score and gambling frequency.

	Model 2		
	IRR	95% CI	*P*
Count component			
Frequency of spending money on gambling activities in the past 12 months			
Once or twice a year (ref.)	1	(1, 1)	(.)
Every 2–3 months	0.907	(0.466, 1.767)	(0.775)
Once a month	1.789	(0.963, 3.323)	(0.066)
Less than once a week, more than once a month	1.370	(0.762, 2.463)	(0.292)
Once a week	2.297	(1.313, 4.018)	(0.004)
2 or more times a week	3.528	(2.040, 6.102)	(0.000)
Age			
18–34 years (ref.)	1	(1, 1)	(.)
35–49 years	1.102	(0.859, 1.414)	(0.445)
50–64 years	0.988	(0.730, 1.336)	(0.936)
65+ years	0.552	(0.358, 0.852)	(0.007)
Sex			
Male (ref.)	1	(1, 1)	(.)
Female	0.580	(0.442, 0.761)	(0.000)
NS‐SEC (occupation)			
Higher managerial and professional occupations (ref.)	1	(1, 1)	(.)
Lower managerial and professional occupations	1.783	(1.127, 2.820)	(0.013)
Intermediate occupations	1.401	(0.844, 2.323)	(0.192)
Small employers and own account workers	1.815	(1.078, 3.055)	(0.025)
Lower supervisory and technical occupations	1.167	(0.677, 2.012)	(0.579)
Semi‐routine occupations	1.853	(1.144, 3.000)	(0.012)
Routine occupations	2.339	(1.408, 3.887)	(0.001)
Never worked and long‐term unemployed	2.795	(0.952, 8.204)	(0.061)
Other	0.896	(0.363, 2.213)	(0.811)
Frequency of alcohol intake in the past 12 months			
Almost every day (ref.)	1	(1, 1)	(.)
Five or six days a week	1.099	(0.582, 2.077)	(0.771)
Three or four days a week	1.035	(0.661, 1.622)	(0.879)
Once or twice a week	1.162	(0.776, 1.741)	(0.467)
Once or twice a month	1.348	(0.863, 2.106)	(0.189)
Once every couple of months	1.354	(0.808, 2.271)	(0.250)
Once or twice a year	1.475	(0.848, 2.566)	(0.169)
Not at all in the last 12 months/non‐drinker	2.331	(1.305, 4.163)	(0.004)
Long‐term mental health disorder			
No (ref.)	1	(1, 1)	(.)
Yes	1.460	(1.044, 2.041)	(0.027)
Index of Multiple Deprivation for England and Scotland			
1—England (least deprived, ref.)	1	(1, 1)	(.)
1—Scotland (least deprived)	1.171	(0.608, 2.255)	(0.638)
2—England	1.081	(0.679, 1.720)	(0.743)
2—Scotland	1.216	(0.712, 2.075)	(0.474)
3—England	1.459	(0.928, 2.292)	(0.101)
3—Scotland	1.065	(0.579, 1.959)	(0.839)
4—England	0.979	(0.624, 1.535)	(0.927)
4—Scotland	0.796	(0.445, 1.426)	(0.444)
5—England (most deprived)	1.226	(0.788, 1.906)	(0.366)
5—Scotland (most deprived)	1.154	(0.680, 1.958)	(0.597)
Zero component			
Frequency of spending money on gambling activities in the past 12 months			
Once or twice a year (ref.)	1	(1, 1)	(.)
Every 2–3 months	0.311	(0.164, 0.592)	(0.000)
Once a month	0.266	(0.152, 0.467)	(0.000)
Less than once a week, more than once a month	0.122	(0.0696, 0.214)	(0.000)
Once a week	0.190	(0.116, 0.314)	(0.000)
2 or more times a week	0.0481	(0.0289, 0.0799)	(0.000)
Age			
18–34 years (ref.)	1	(1, 1)	(.)
35–49 years	3.551	(2.670, 4.724)	(0.000)
50–64 years	7.904	(5.739, 10.89)	(0.000)
65+ years	17.35	(11.39, 26.42)	(0.000)
Sex			
Male (ref.)	1	(1, 1)	(.)
Female	3.195	(2.467, 4.137)	(0.000)
NS‐SEC (occupation)			
Higher managerial and professional occupations (ref.)	1	(1, 1)	(.)
Lower managerial and professional occupations	0.919	(0.580, 1.455)	(0.717)
Intermediate occupations	0.780	(0.463, 1.314)	(0.350)
Small employers and own account workers	0.725	(0.425, 1.237)	(0.239)
Lower supervisory and technical occupations	0.864	(0.485, 1.537)	(0.618)
Semi‐routine occupations	0.794	(0.485, 1.299)	(0.359)
Routine occupations	0.878	(0.531, 1.450)	(0.611)
Never worked and long‐term unemployed	1.006	(0.295, 3.430)	(0.992)
Other	0.0551	(0.00339, 0.895)	(0.042)
Frequency of alcohol intake in the past 12 months			
Almost every day (ref.)	1	(1, 1)	(.)
Five or six days a week	0.941	(0.486, 1.822)	(0.856)
Three or four days a week	0.851	(0.531, 1.363)	(0.502)
Once or twice a week	1.058	(0.688, 1.626)	(0.798)
Once or twice a month	1.503	(0.939, 2.407)	(0.089)
Once every couple of months	1.066	(0.622, 1.825)	(0.817)
Once or twice a year	1.281	(0.737, 2.225)	(0.380)
Not at all in the last 12 months/non‐drinker	1.742	(1.007, 3.011)	(0.047)
Long‐term mental health disorder			
No (ref.)	1	(1, 1)	(.)
Yes	0.529	(0.367, 0.762)	(0.001)
Index of Multiple Deprivation for England and Scotland			
1—England (least deprived, ref.)	1	(1, 1)	(.)
1—Scotland (least deprived)	1.773	(0.980, 3.210)	(0.059)
2—England	0.856	(0.538, 1.363)	(0.512)
2—Scotland	1.374	(0.816, 2.312)	(0.232)
3—England	1.194	(0.770, 1.851)	(0.428)
3—Scotland	1.555	(0.876, 2.761)	(0.132)
4—England	0.776	(0.489, 1.233)	(0.284)
4—Scotland	1.271	(0.702, 2.302)	(0.429)
5—England (most deprived)	0.582	(0.370, 0.915)	(0.019)
5—Scotland (most deprived)	0.799	(0.463, 1.379)	(0.420)
Observations	16 648		
ln(*α*)	2.782	(2.211, 3.502)	(0.000)

Abbreviations: PGSI = problem gambling severity index; NS‐SEC = National Statistics Socio‐economic Classification.

### The association between gambling frequency and PGSI score controlling only for age, sex and deprivation (reduced model)

The results for the model with the reduced number of control variables appeared similar to the main results, with minor qualitative differences in the IRRs but with overlapping 95% CIs. The full results of these analyses can be found in Table S3.

### Sensitivity analysis—excluding people who only gamble on lotteries

The sample characteristics for the subsample (excluding those who solely gambled on lotteries) can be seen in Table S4 along with the characteristics of those excluded in Table S5. The results of the multinomial logistic regression looking at PGSI categories can be seen in Table S6 (model 1b). The results were similar as those for the whole sample analysis except when looking at gambling frequency and its association with the high‐risk category, where the results were no longer significant. The sample sizes for these groups were very small (Table S4). The results of the zero‐inflated negative binomial can be seen in Table S7. Qualitatively the results appear similar, with the 95% CIs overlapping with those from the main analysis.

### Hypothetical modelling of a policy to reduce gambling frequency

Table 5 shows the impact of a hypothetical policy that reduced the gambling frequency of a subsample who gambled above the lower risk gambling guidelines to a gambling frequency in line with the guidelines when using the multinomial logistic regression model to predict PGSI category. Approximately 10% of the population moved down into the no‐risk category from the higher risk categories.

**TABLE 5:** The distribution of the high‐frequency subsample across PGSI categories before and after reducing gambling frequency to align with the lower‐risk gambling guidelines.

PGSI category	Actual distribution of the sample population	Predicted distribution of the population before applying the guidelines	Predicted distribution of the population after applying the guidelines
		**Multinomial logistic regression with gambling frequency as categorical**	
No risk	83.2%	83.2%	92.9%
Low risk	8.6%	8.8%	4.1%
Moderate risk	5.4%	5.1%	1.7%
High risk	2.9%	2.9%	1.2%

Abbreviation: PGSI = problem gambling severity index.

**FIGURE 3:**
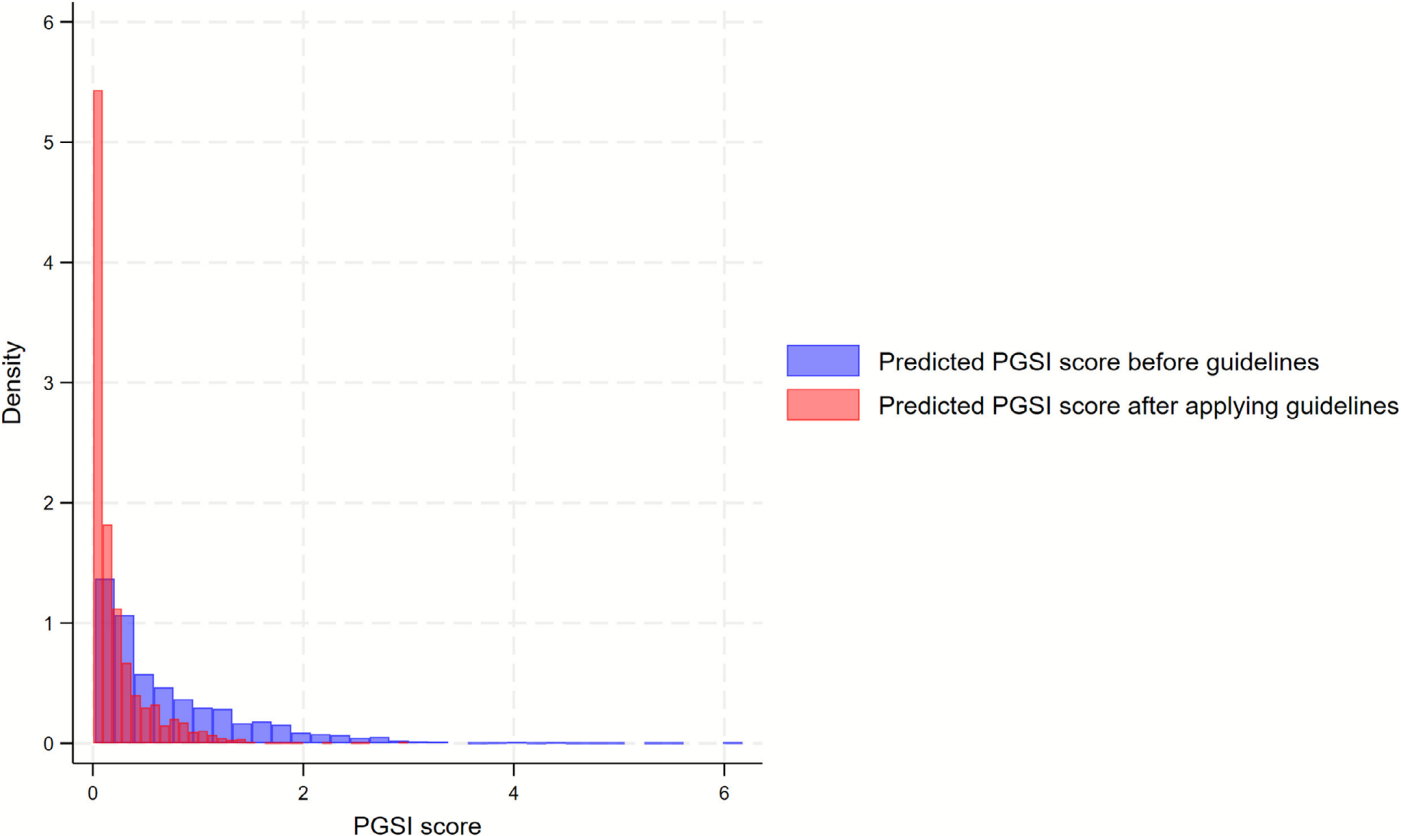
Distribution of problem gambling severity index (PGSI) scores within the high‐frequency gambling subsample (individuals who gamble two or more times per week), shown both before and after a hypothetical reduction in gambling frequency to once per week, in line with lower risk gambling guidelines.

Figure 3 shows the distribution of PGSI scores, predicted using the zero‐inflated negative binomial model, before and after the guidelines. The distribution shifted left, indicating that the overall PGSI scores might have decreased.

## DISCUSSION

This study quantified the association between gambling frequency and the risk of gambling‐related harm. We found a significant positive association between gambling frequency and having a higher PGSI score or the likelihood of being in a more severe PGSI category. This is consistent with relationships described in the international literature [14], and addresses a gap in the evidence by calculating RRRs and IRRs for the association between gambling frequency and risk of harm, which can be used in health economic modelling. While this study uses data from England and Scotland, the observed positive association between gambling frequency and the risk of harm, as measured by PGSI, is likely to be relevant globally. In future research, it would be beneficial to test our models using alternative data from data sources other than the HSE or the SHeS.

We also demonstrated how the results of this analysis could be used to predict the impact of gambling policies that aim to change gambling frequency. Future research could implement these results into a larger public health economic model of gambling by linking PGSI category to the prevalence of gambling‐related harms, and associate a cost with these, to estimate the cost‐effectiveness of policies that impact gambling frequency. For example, there is evidence to support a dose–response relationship between gambling advertising and gambling frequency [49]. Therefore, if we knew the impact of advertising on someone's gambling frequency, we could use the results of this model to link this to risk of harm (PGSI category) and understand how this would impact the prevalence of gambling‐related harms at the population level.

While this analysis benefits from a large, representative sample (with six waves of the HSE and SHeS combined) and a rich data set allowing for suitable control variables to be included, limitations do exist. Nearly 15% of the sample were excluded owing to incomplete data. We assumed these data were missing completely at random and conducted a complete case analysis. If this assumption does not hold this could introduce bias through systematic differences between the included and excluded cases. In addition, the data set might not reflect current gambling behaviour as the data were collected between 2015 and 2018. A further limitation is that this study used cross‐sectional data that contain one single time point for both gambling frequency and the PGSI. This temporal snapshot means a recent increase in gambling frequency might not yet be reflected in the corresponding PGSI score. However, the gambling frequency question asked about frequency in the last 12 months, so it would be expected that responders used their average frequency over the last 12 months to answer the question. The cross‐sectional nature also means we cannot state there is a causal relationship between this measure of gambling frequency and the risk of gambling‐related harm. An additional limitation is that the data were collected via self‐report, which has been found to be unreliable in the context of gambling. A study that compared self‐reported data with data from gambling companies reported that only 7% of people reported their gambling frequency accurately to within a 10% margin of their actual gambling frequency [50]. Additionally, in our data the highest category of the gambling frequency measure, ‘two or more times weekly’, likely underestimates high‐frequency gambling, as daily gambling is common. In some countries (e.g. Norway) it is possible to use expenditure data from gambling operators to overcome the limitations of self‐report and provide a more accurate and detailed understanding of gambling behaviour [13]. The inability to disaggregate gambling frequency by specific activity type also represents a limitation. Our analysis excluded those who only gambled on lotteries, and found very little difference in the model results, which contrasts with the work reported by Currie *et al*., who found that playing the lottery was low risk at all levels of frequency and suggested that any guidelines on frequency might need to be different for each type of gambling activity [46]. Independent UK researchers currently rely on self‐reported, cross‐sectional surveys. A centralised, anonymised and ideally longitudinal data repository of customer gambling behaviour, combined with measures like the PGSI, would significantly improve future research.

This study used gambling frequency as a measure of gambling behaviour owing to the availability of data and the concerns around the reliability of estimates of gambling spending. However, we know that gambling frequency is only one aspect of gambling behaviour, and that people can gamble at high frequencies and still experience low levels of gambling‐related harm. This could be the result of many factors, such as gambling low amounts in relation to income or other environmental or social factors that protect people from harm. Therefore, future research could assess which measures of gambling behaviours are best able to predict the risk of harm, or even research the possibility of a composite measure including multiple measures. Research towards developing the Canadian lower risk gambling guidelines looked at multiple measures of gambling behaviour, including frequency, the number of types of gambling and the percentage of income spent, but treated these as separate entities [51]. It would be useful to investigate how these interact and whether a composite measure could be used to accurately predict someone's risk of gambling‐related harm. For example, it is likely that someone who gambles once a week but spends 1% of their income on gambling might be at lower risk of harm compared with someone who gambles twice a month but gambles 10% of their income. Reaching a consensus on how best to measure gambling behaviour to predict risk of harm would help direct spending on surveys to make sure the most appropriate questions are included.

In conclusion, these analyses found a significant association between gambling frequency and risk of gambling‐related harm, defined using the PGSI. We have demonstrated how these results could be used to predict the impact of policies that result in a change in gambling frequency. Future research should expand on this to understand the impact not only on PGSI but on the prevalence of gambling‐related harms, and continue to investigate which measure of gambling behaviour most accurately predicts the risk of gambling‐related harm.

## AUTHOR CONTRIBUTIONS

**Esther Moore:** Conceptualization (lead); data curation (lead); formal analysis (lead); funding acquisition (lead); investigation (lead); methodology (lead); project administration (lead); writing—original draft (lead); writing—review and editing (lead). **Robert Pryce:** Conceptualization (supporting); formal analysis (supporting); methodology (supporting); project administration (supporting); supervision (lead); writing—original draft (supporting). **Hazel Squires:** Conceptualization (supporting); formal analysis (supporting); methodology (supporting); project administration (supporting); supervision (supporting); writing—original draft (supporting). **Elizabeth Goyder:** Conceptualization (supporting); methodology (supporting); supervision (supporting); writing—original draft (supporting).

## DECLARATION OF INTERESTS

The authors declare no competing interests.

## Supporting information


**Table S1.** A comparison of the negative binomial model and zero‐inflated negative binomial model for Model 2 for looking at the relationship between PGSI score and gambling frequency.
**Table S2.** The results for model 1a, a multinomial logistic regression looking at the relationship between PGSI category and gambling frequency with a reduced set of control variable.
**Table S3.** The results for model 2a, a zero‐inflated negative binomial model looking at the relationship between PGSI score and gambling frequency with a reduced set of control variables.
**Table S4.** The descriptive statistics of the analytic sample for the analysis excluding those who only gambled on a lottery.
**Table S5.** The descriptive statistics of those who only gambled on a lottery and were excluded from the analysis.
**Table S6**. The results of the multinomial logistic regressions looking at the relationship between PGSI category and gambling frequency in a sample who do not solely gamble on lotteries.
**Table S7**. The results of the zero‐inflated negative binomial models looking at the relationship between PGSI score and gambling frequency in sample who do not solely gamble on lotteries.

## Data Availability

The data that support the findings of this study are openly available via the UK Data Service at https://doi.org/10.5255/UKDA-Series-2000047 and https://doi.org/10.5255/UKDA-Series-2000021.
