# Supplementary material for: The association between gambling frequency and risk of harm: Analysis using health survey data from England and Scotland
Source: Addiction. 2026 Feb 17;121(6):1594–610. doi: 10.1111/add.70344 (PMC13155269; doi:10.1111/add.70344)
Supplement: Supplementary file 1 — Table S1. A comparison of the negative binomial model and zero‐inflated negative binomial model for Model 2 for looking at the relationship between PGSI score and gambling frequency. Table S2. The results for model 1a, a multinomial logistic regression looking at the relationship between PGSI category and gambling frequency with a reduced set of control variable. Table S3. The results for model 2a, a zero‐inflated negative binomial model looking at the relationship between PGSI score and gambling frequency with a reduced set of control variables. Table S4. The descriptive statistics of the analytic sample for the analysis excluding those who only gambled on a lottery. Table S5. The descriptive statistics of those who only gambled on a lottery and were excluded from the analysis. Table S6. The results of the multinomial logistic regressions looking at the relationship between PGSI category and gambling frequency in a sample who do not solely gamble on lotteries. Table S7. The results of the zero‐inflated negative binomial models looking at the relationship between PGSI score and gambling frequency in sample who do not solely gamble on lotteries. [file ADD-121-1594-s001.docx]

**Table S1** A comparison of the negative binomial model and zero-inflated negative binomial model for Model 2 for looking at the relationship between PGSI score and gambling frequency. PGSI= Problem Gambling Severity Index.

|  | Model 2 | |
| --- | --- | --- |
|  | Negative binomial model | Zero-inflated negative binomial |
| Akaike Information Criterion (AIC) | 10146.535 | 9788.555 |
| Bayesian Information Criterion (BIC) | 10424.457 | 10305.798 |
|  |  |  |

**Table S2** The results for Model 1a  a multinomial logistic regression looking at the relationship between PGSI category and gambling frequency with a reduced set of control variable. PGSI = Problem Gambling Severity Index.

|  | Model 1a | | |
| --- | --- | --- | --- |
|  | Relative risk ratio | 95% confidence interval | P-value |
| PGSI Category: No-Risk | Reference group | | |
| PGSI Category: Low-Risk |  |  |  |
| *Frequency of spending money on gambling activities in the past 12 months* |  |  |  |
| Ref: Once or twice a year | 1 | [1,1] | (.) |
| Every 2-3 months | 2.548 | [1.667,3.893] | (0.000) |
| Once a month | 3.835 | [2.545,5.779] | (0.000) |
| Less than once a week, more than once a month | 7.074 | [4.820,10.38] | (0.000) |
| Once a week | 5.163 | [3.579,7.448] | (0.000) |
| 2 or more times a week | 15.01 | [10.38,21.71] | (0.000) |
| *Age* |  |  |  |
| Ref: Age 18-34 | 1 | [1,1] | (.) |
| Age 35-49 | 0.383 | [0.312,0.470] | (0.000) |
| Age 50-64 | 0.197 | [0.156,0.249] | (0.000) |
| Age 65+ | 0.0868 | [0.0629,0.120] | (0.000) |
| *Sex* |  |  |  |
| Ref: Male | 1 | [1,1] | (.) |
| Female | 0.339 | [0.281,0.409] | (0.000) |
| *Index of Multiple Deprivation for England and Scotland* |  |  |  |
| Least deprived- England | 1 | [1,1] | (.) |
| Least deprived- Scotland | 0.645 | [0.415,1.003] | (0.052) |
| 2- England | 1.066 | [0.771,1.472] | (0.699) |
| 2- Scotland | 0.697 | [0.465,1.045] | (0.081) |
| 3- England | 0.806 | [0.577,1.126] | (0.207) |
| 3- Scotland | 0.581 | [0.372,0.908] | (0.017) |
| 4- England | 1.033 | [0.751,1.423] | (0.840) |
| 4- Scotland | 0.719 | [0.472,1.096] | (0.125) |
| Most deprived- England | 1.301 | [0.954,1.774] | (0.097) |
| Most deprived- Scotland | 0.925 | [0.618,1.385] | (0.706) |
| PGSI Category: Moderate-Risk |  |  |  |
| *Frequency of spending money on gambling activities in the past 12 months* |  |  |  |
| Ref: Once or twice a year | 1 | [1,1] | (.) |
| Every 2-3 months | 2.383 | [1.014,5.602] | (0.046) |
| Once a month | 3.402 | [1.480,7.818] | (0.004) |
| Less than once a week, more than once a month | 5.400 | [2.456,11.87] | (0.000) |
| Once a week | 8.119 | [4.003,16.46] | (0.000) |
| 2 or more times a week | 38.64 | [19.33,77.23] | (0.000) |
| *Age* |  |  |  |
| Ref: Age 18-34 | 1 | [1,1] | (.) |
| Age 35-49 | 0.347 | [0.252,0.479] | (0.000) |
| Age 50-64 | 0.160 | [0.111,0.230] | (0.000) |
| Age 65+ | 0.0527 | [0.0302,0.0919] | (0.000) |
| *Sex* |  |  |  |
| Ref: Male | 1 | [1,1] | (.) |
| Female | 0.298 | [0.217,0.408] | (0.000) |
| *Index of Multiple Deprivation for England and Scotland* |  |  |  |
| Least deprived- England | 1 | [1,1] | (.) |
| Least deprived- Scotland | 0.769 | [0.326,1.813] | (0.548) |
| 2- England | 1.171 | [0.608,2.253] | (0.637) |
| 2- Scotland | 0.898 | [0.421,1.916] | (0.781) |
| 3- England | 1.890 | [1.052,3.396] | (0.033) |
| 3- Scotland | 1.149 | [0.555,2.381] | (0.708) |
| 4- England | 1.562 | [0.855,2.853] | (0.147) |
| 4- Scotland | 0.944 | [0.433,2.057] | (0.884) |
| Most deprived- England | 2.998 | [1.713,5.247] | (0.000) |
| Most deprived- Scotland | 2.308 | [1.220,4.365] | (0.010) |
| PGSI Category: High-Risk |  |  |  |
| *Frequency of spending money on gambling activities in the past 12 months* |  |  |  |
| Ref: Once or twice a year | 1 | [1,1] | (.) |
| Every 2-3 months | 4.870 | [0.506,46.91] | (0.171) |
| Once a month | 21.94 | [2.824,170.4] | (0.003) |
| Less than once a week, more than once a month | 19.91 | [2.515,157.6] | (0.005) |
| Once a week | 31.16 | [4.228,229.6] | (0.001) |
| 2 or more times a week | 164.1 | [22.59,1191.4] | (0.000) |
| *Age* |  |  |  |
| Ref: Age 18-34 | 1 | [1,1] | (.) |
| Age 35-49 | 0.447 | [0.285,0.701] | (0.000) |
| Age 50-64 | 0.211 | [0.129,0.347] | (0.000) |
| Age 65+ | 0.0474 | [0.0200,0.113] | (0.000) |
| *Sex* |  |  |  |
| Ref: Male | 1 | [1,1] | (.) |
| Female | 0.182 | [0.108,0.306] | (0.000) |
| *Index of Multiple Deprivation for England and Scotland* |  |  |  |
| Least deprived- England | 1 | [1,1] | (.) |
| Least deprived- Scotland | 0.544 | [0.109,2.728] | (0.460) |
| 2- England | 1.773 | [0.648,4.851] | (0.265) |
| 2- Scotland | 2.337 | [0.850,6.426] | (0.100) |
| 3- England | 2.372 | [0.924,6.090] | (0.073) |
| 3- Scotland | 1.642 | [0.543,4.966] | (0.380) |
| 4- England | 2.459 | [0.963,6.281] | (0.060) |
| 4- Scotland | 0.971 | [0.270,3.493] | (0.964) |
| Most deprived- England | 4.313 | [1.759,10.57] | (0.001) |
| Most deprived- Scotland | 3.298 | [1.228,8.852] | (0.018) |
| Observations | 16648 |  |  |
| Pseudo R^2^ | 0.177 |  |  |

**Table S3** The results for Model 2a a zero-inflated negative binomial model looking at the relationship between PGSI score and gambling frequency with a reduced set of control variables

|  | Model 2a | | |
| --- | --- | --- | --- |
|  | Incidence Rate Ratio | 95% confidence interval | P-value |
| **Count component** |  |  |  |
| *Frequency of spending money on gambling activities in the past 12 months* |  |  |  |
| Ref: Once or twice a year | 1 | [1,1] | (.) |
| Every 2-3 months | 0.767 | [0.395,1.490] | (0.434) |
| Once a month | 1.579 | [0.845,2.950] | (0.152) |
| Less than once a week, more than once a month | 1.453 | [0.809,2.611] | (0.211) |
| Once a week | 2.233 | [1.266,3.941] | (0.006) |
| 2 or more times a week | 3.440 | [1.976,5.988] | (0.000) |
| *Age* |  |  |  |
| Ref: Age 18-34 | 1 | [1,1] | (.) |
| Age 35-49 | 1.224 | [0.954,1.571] | (0.112) |
| Age 50-64 | 1.103 | [0.822,1.480] | (0.512) |
| Age 65+ | 0.638 | [0.418,0.974] | (0.037) |
| *Sex* |  |  |  |
| Ref: Male | 1 | [1,1] | (.) |
| Female | 0.647 | [0.498,0.841] | (0.001) |
| *Index of Multiple Deprivation for England and Scotland* |  |  |  |
| Ref: Least deprived- England | 1 | [1,1] | (.) |
| Least deprived- Scotland | 1.146 | [0.605,2.171] | (0.675) |
| 2- England | 1.213 | [0.768,1.915] | (0.408) |
| 2- Scotland | 1.415 | [0.832,2.404] | (0.200) |
| 3- England | 1.805 | [1.155,2.818] | (0.009) |
| 3- Scotland | 1.549 | [0.874,2.745] | (0.134) |
| 4- England | 1.280 | [0.832,1.969] | (0.262) |
| 4- Scotland | 0.954 | [0.536,1.697] | (0.872) |
| Most deprived- England | 1.840 | [1.221,2.771] | (0.004) |
| Most deprived- Scotland | 1.543 | [0.938,2.538] | (0.088) |
| **Zero component** |  |  |  |
| *Frequency of spending money on gambling activities in the past 12 months* |  |  |  |
| Ref: Once or twice a year | 1 | [1,1] | (.) |
| Every 2-3 months | 0.267 | [0.139,0.511] | (0.000) |
| Once a month | 0.251 | [0.142,0.442] | (0.000) |
| Less than once a week, more than once a month | 0.126 | [0.0729,0.218] | (0.000) |
| Once a week | 0.184 | [0.112,0.301] | (0.000) |
| 2 or more times a week | 0.0448 | [0.0270,0.0743] | (0.000) |
| *Age* |  |  |  |
| Ref: Age 18-34 | 1 | [1,1] | (.) |
| Age 35-49 | 3.925 | [2.930,5.257] | (0.000) |
| Age 50-64 | 8.476 | [6.151,11.68] | (0.000) |
| Age 65+ | 20.01 | [13.18,30.39] | (0.000) |
| *Sex* |  |  |  |
| Ref: Male | 1 | [1,1] | (.) |
| Female | 3.216 | [2.511,4.120] | (0.000) |
| *Index of Multiple Deprivation for England and Scotland* |  |  |  |
| Ref: Least deprived- England | 1 | [1,1] | (.) |
| Least deprived- Scotland | 1.807 | [0.989,3.302] | (0.054) |
| 2- England | 0.885 | [0.553,1.416] | (0.610) |
| 2- Scotland | 1.496 | [0.886,2.527] | (0.132) |
| 3- England | 1.233 | [0.795,1.914] | (0.349) |
| 3- Scotland | 1.767 | [1.021,3.057] | (0.042) |
| 4- England | 0.830 | [0.528,1.306] | (0.421) |
| 4- Scotland | 1.284 | [0.704,2.343] | (0.414) |
| Most deprived- England | 0.626 | [0.405,0.967] | (0.035) |
| Most deprived- Scotland | 0.805 | [0.472,1.373] | (0.426) |
| Observations | 16648 |  |  |
| ln(α) | 3.194 | [2.549,4.003] | (0.000) |

**Table S4** The descriptive statistics of the analytic sample for the analysis excluding those who only gambled on a lottery.

|  | PGSI* Category | | | | | |
| --- | --- | --- | --- | --- | --- | --- |
|  | No Risk  N (%) | Low Risk  N (%) | Moderate Risk  N (%) | High Risk  N (%) | Total  N (%) | Chi-squared test |
| Total | 8,478 (91.0%) | 537 (5.8%) | 214 (2.3%) | 91 (1.0%) | 9,320 (100.0%) |  |
| *Sex* |  |  |  |  |  |  |
| Male | 3,986 (47.0%) | 413 (76.9%) | 170 (79.4%) | 80 (87.9%) | 4,649 (49.9%) | <0.001 |
| Female | 4,492 (53.0%) | 124 (23.1%) | 44 (20.6%) | 11 (12.1%) | 4,671 (50.1%) |  |
| *Age* |  |  |  |  |  |  |
| Age 18-34 | 2,487 (29.3%) | 263 (49.0%) | 103 (48.1%) | 41 (45.1%) | 2,894 (31.1%) | <0.001 |
| Age 35-49 | 2,583 (30.5%) | 145 (27.0%) | 63 (29.4%) | 28 (30.8%) | 2,819 (30.2%) |  |
| Age 50-64 | 2,117 (25.0%) | 97 (18.1%) | 37 (17.3%) | 18 (19.8%) | 2,269 (24.3%) |  |
| Age 65+ | 1,291 (15.2%) | 32 (6.0%) | 11 (5.1%) | 4 (4.4%) | 1,338 (14.4%) |  |
| *Index of Multiple Deprivation for England and Scotland* |  |  |  |  |  |  |
| Ref: Least deprived- England | 1,033 (12.2%) | 67 (12.5%) | 17 (7.9%) | 4 (4.4%) | 1,121 (12.0%) | <0.001 |
| Least deprived- Scotland | 583 (6.9%) | 26 (4.8%) | 7 (3.3%) | 2 (2.2%) | 618 (6.6%) |  |
| 2- England | 1,134 (13.4%) | 81 (15.1%) | 20 (9.3%) | 10 (11.0%) | 1,245 (13.4%) |  |
| 2- Scotland | 697 (8.2%) | 37 (6.9%) | 10 (4.7%) | 8 (8.8%) | 752 (8.1%) |  |
| 3- England | 1,205 (14.2%) | 67 (12.5%) | 35 (16.4%) | 12 (13.2%) | 1,319 (14.2%) |  |
| 3- Scotland | 626 (7.4%) | 24 (4.5%) | 11 (5.1%) | 6 (6.6%) | 667 (7.2%) |  |
| 4- England | 1,072 (12.6%) | 82 (15.3%) | 29 (13.6%) | 14 (15.4%) | 1,197 (12.8%) |  |
| 4- Scotland | 561 (6.6%) | 34 (6.3%) | 10 (4.7%) | 3 (3.3%) | 608 (6.5%) |  |
| Most deprived- England | 1,055 (12.4%) | 84 (15.6%) | 52 (24.3%) | 21 (23.1%) | 1,212 (13.0%) |  |
| Most deprived- Scotland | 512 (6.0%) | 35 (6.5%) | 23 (10.7%) | 11 (12.1%) | 581 (6.2%) |  |
| *NS-SEC^±^ (Occupation)* |  |  |  |  |  |  |
| Higher managerial and professional occupations | 1,024 (12.1%) | 52 (9.7%) | 15 (7.0%) | 2 (2.2%) | 1,093 (11.7%) | <0.001 |
| Lower managerial and professional occupations | 2,144 (25.3%) | 126 (23.5%) | 49 (22.9%) | 20 (22.0%) | 2,339 (25.1%) |  |
| Intermediate occupations | 1,252 (14.8%) | 67 (12.5%) | 19 (8.9%) | 5 (5.5%) | 1,343 (14.4%) |  |
| Small employers and own account workers | 715 (8.4%) | 51 (9.5%) | 24 (11.2%) | 8 (8.8%) | 798 (8.6%) |  |
| Lower supervisory and technical occupations | 653 (7.7%) | 49 (9.1%) | 22 (10.3%) | 7 (7.7%) | 731 (7.8%) |  |
| Semi-routine occupations | 1,530 (18.0%) | 97 (18.1%) | 40 (18.7%) | 20 (22.0%) | 1,687 (18.1%) |  |
| Routine occupations | 1,084 (12.8%) | 79 (14.7%) | 42 (19.6%) | 27 (29.7%) | 1,232 (13.2%) |  |
| Never worked and long term unemployed | 46 (0.5%) | 6 (1.1%) | 0 (0.0%) | 2 (2.2%) | 54 (0.6%) |  |
| Other | 30 (0.4%) | 10 (1.9%) | 3 (1.4%) | 0 (0.0%) | 43 (0.5%) |  |
| *Long-term mental health disorder* |  |  |  |  |  |  |
| No | 7,849 (92.6%) | 490 (91.2%) | 193 (90.2%) | 73 (80.2%) | 8,605 (92.3%) | <0.001 |
| Yes | 629 (7.4%) | 47 (8.8%) | 21 (9.8%) | 18 (19.8%) | 715 (7.7%) |  |
| *Frequency drunk alcohol in past 12 months* |  |  |  |  |  |  |
| Almost every day | 593 (7.0%) | 35 (6.5%) | 23 (10.7%) | 7 (7.7%) | 658 (7.1%) | 0.351 |
| Five or six days a week | 355 (4.2%) | 24 (4.5%) | 10 (4.7%) | 2 (2.2%) | 391 (4.2%) |  |
| Three or four days a week | 1,282 (15.1%) | 104 (19.4%) | 37 (17.3%) | 10 (11.0%) | 1,433 (15.4%) |  |
| Once or twice a week | 2,891 (34.1%) | 180 (33.5%) | 79 (36.9%) | 35 (38.5%) | 3,185 (34.2%) |  |
| Once or twice a month | 1,485 (17.5%) | 86 (16.0%) | 27 (12.6%) | 15 (16.5%) | 1,613 (17.3%) |  |
| Once every couple of months | 761 (9.0%) | 48 (8.9%) | 15 (7.0%) | 10 (11.0%) | 834 (8.9%) |  |
| Once or twice a year | 629 (7.4%) | 37 (6.9%) | 13 (6.1%) | 5 (5.5%) | 684 (7.3%) |  |
| Not at all in the last 12 months/Non-drinker | 482 (5.7%) | 23 (4.3%) | 10 (4.7%) | 7 (7.7%) | 522 (5.6%) |  |
| *Frequency of spending money on any gambling activities* |  |  |  |  |  |  |
| 2 or more times a week | 937 (11.1%) | 167 (31.1%) | 107 (50.0%) | 56 (61.5%) | 1,267 (13.6%) | <0.001 |
| Once a week | 2,032 (24.0%) | 132 (24.6%) | 54 (25.2%) | 20 (22.0%) | 2,238 (24.0%) |  |
| Less than once a week, more than once a month | 892 (10.5%) | 98 (18.2%) | 18 (8.4%) | 4 (4.4%) | 1,012 (10.9%) |  |
| Once a month | 921 (10.9%) | 58 (10.8%) | 13 (6.1%) | 9 (9.9%) | 1,001 (10.7%) |  |
| Every 2-3 months | 1,222 (14.4%) | 49 (9.1%) | 13 (6.1%) | 2 (2.2%) | 1,286 (13.8%) |  |
| Once or twice a year | 2,474 (29.2%) | 33 (6.1%) | 9 (4.2%) | 0 (0.0%) | 2,516 (27.0%) |  |
| Mean PGSI score (standard error) | 0.000 (0.000) | 1.272 (0.445) | 4.364 (1.352) | 13.604 (5.835) | 0.306 (1.621) | NA |

^*^ PGSI = Problem Gambling Severity Index

^±^ NS-SEC = National Statistics Socio-economic classification

**Table S5 The descriptive statistics of those who only gambled on a lottery and were excluded from the analysis.^†^**

|  | PGSI* Category | | | | | |
| --- | --- | --- | --- | --- | --- | --- |
|  | No Risk  N (%) | Low Risk  N (%) | Moderate Risk  N (%) | High Risk  N (%) | Total  N (%) | Chi-squared test |
| Total | 6,056 (99.3%) | 27 (0.4%) | 8 (0.1%) | 7 (0.1%) | 6,098 (100.0%) |  |
| *Sex* |  |  |  |  |  |  |
| Male | 2,737 (45.2%) | 12 (44.4%) | 4 (50.0%) | 4 (57.1%) | 2,757 (45.2%) | 0.923 |
| Female | 3,319 (54.8%) | 15 (55.6%) | 4 (50.0%) | 3 (42.9%) | 3,341 (54.8%) |  |
| *Age* |  |  |  |  |  |  |
| Age 18-34 | 526 (8.7%) | 4 (14.8%) | 2 (25.0%) | 0 (0.0%) | 532 (8.7%) | 0.051 |
| Age 35-49 | 1,422 (23.5%) | 12 (44.4%) | 2 (25.0%) | 1 (14.3%) | 1,437 (23.6%) |  |
| Age 50-64 | 2,110 (34.8%) | 6 (22.2%) | 3 (37.5%) | 5 (71.4%) | 2,124 (34.8%) |  |
| Age 65+ | 1,998 (33.0%) | 5 (18.5%) | 1 (12.5%) | 1 (14.3%) | 2,005 (32.9%) |  |
| *Index of Multiple Deprivation for England and Scotland* |  |  |  |  |  |  |
| Ref: Least deprived- England | 937 (15.5%) | 6 (22.2%) | 0 (0.0%) | 1 (14.3%) | 944 (15.5%) | 0.049 |
| Least deprived- Scotland | 471 (7.8%) | 3 (11.1%) | 0 (0.0%) | 0 (0.0%) | 474 (7.8%) |  |
| 2- England | 897 (14.8%) | 2 (7.4%) | 0 (0.0%) | 0 (0.0%) | 899 (14.7%) |  |
| 2- Scotland | 507 (8.4%) | 0 (0.0%) | 2 (25.0%) | 1 (14.3%) | 510 (8.4%) |  |
| 3- England | 889 (14.7%) | 1 (3.7%) | 0 (0.0%) | 3 (42.9%) | 893 (14.6%) |  |
| 3- Scotland | 444 (7.3%) | 1 (3.7%) | 2 (25.0%) | 0 (0.0%) | 447 (7.3%) |  |
| 4- England | 762 (12.6%) | 3 (11.1%) | 1 (12.5%) | 1 (14.3%) | 767 (12.6%) |  |
| 4- Scotland | 343 (5.7%) | 1 (3.7%) | 1 (12.5%) | 0 (0.0%) | 345 (5.7%) |  |
| Most deprived- England | 565 (9.3%) | 8 (29.6%) | 2 (25.0%) | 1 (14.3%) | 576 (9.4%) |  |
| Most deprived- Scotland | 241 (4.0%) | 2 (7.4%) | 0 (0.0%) | 0 (0.0%) | 243 (4.0%) |  |
| *NS-SEC^±^ (Occupation)* |  |  |  |  |  |  |
| Higher managerial and professional occupations | 835 (13.8%) | 6 (22.2%) | 2 (25.0%) | 0 (0.0%) | 843 (13.8%) | 0.935 |
| Lower managerial and professional occupations | 1,634 (27.0%) | 5 (18.5%) | 2 (25.0%) | 2 (28.6%) | 1,643 (26.9%) |  |
| Intermediate occupations | 953 (15.7%) | 2 (7.4%) | 0 (0.0%) | 1 (14.3%) | 956 (15.7%) |  |
| Small employers and own account workers | 573 (9.5%) | 1 (3.7%) | 1 (12.5%) | 1 (14.3%) | 576 (9.4%) |  |
| Lower supervisory and technical occupations | 426 (7.0%) | 1 (3.7%) | 1 (12.5%) | 0 (0.0%) | 428 (7.0%) |  |
| Semi-routine occupations | 987 (16.3%) | 7 (25.9%) | 1 (12.5%) | 1 (14.3%) | 996 (16.3%) |  |
| Routine occupations | 626 (10.3%) | 5 (18.5%) | 1 (12.5%) | 2 (28.6%) | 634 (10.4%) |  |
| Never worked and long term unemployed | 14 (0.2%) | 0 (0.0%) | 0 (0.0%) | 0 (0.0%) | 14 (0.2%) |  |
| Other | 8 (0.1%) | 0 (0.0%) | 0 (0.0%) | 0 (0.0%) | 8 (0.1%) |  |
| *Long-term mental health disorder* |  |  |  |  |  |  |
| No | 5,709 (94.3%) | 21 (77.8%) | 6 (75.0%) | 6 (85.7%) | 5,742 (94.2%) | <0.001 |
| Yes | 347 (5.7%) | 6 (22.2%) | 2 (25.0%) | 1 (14.3%) | 356 (5.8%) |  |
| *Frequency drunk alcohol in past 12 months* |  |  |  |  |  |  |
| Almost every day | 598 (9.9%) | 4 (14.8%) | 0 (0.0%) | 0 (0.0%) | 602 (9.9%) | 0.719 |
| Five or six days a week | 293 (4.8%) | 1 (3.7%) | 0 (0.0%) | 0 (0.0%) | 294 (4.8%) |  |
| Three or four days a week | 941 (15.5%) | 3 (11.1%) | 1 (12.5%) | 1 (14.3%) | 946 (15.5%) |  |
| Once or twice a week | 1,820 (30.1%) | 10 (37.0%) | 1 (12.5%) | 1 (14.3%) | 1,832 (30.0%) |  |
| Once or twice a month | 873 (14.4%) | 4 (14.8%) | 2 (25.0%) | 2 (28.6%) | 881 (14.4%) |  |
| Once every couple of months | 496 (8.2%) | 2 (7.4%) | 1 (12.5%) | 0 (0.0%) | 499 (8.2%) |  |
| Once or twice a year | 541 (8.9%) | 0 (0.0%) | 1 (12.5%) | 1 (14.3%) | 543 (8.9%) |  |
| Not at all in the last 12 months/Non-drinker | 494 (8.2%) | 3 (11.1%) | 2 (25.0%) | 2 (28.6%) | 501 (8.2%) |  |
| *Frequency of spending money on any gambling activities* |  |  |  |  |  |  |
| 2 or more times a week | 741 (12.2%) | 8 (29.6%) | 1 (12.5%) | 0 (0.0%) | 750 (12.3%) | 0.126 |
| Once a week | 2,105 (34.8%) | 8 (29.6%) | 5 (62.5%) | 4 (57.1%) | 2,122 (34.8%) |  |
| Less than once a week, more than once a month | 520 (8.6%) | 3 (11.1%) | 1 (12.5%) | 2 (28.6%) | 526 (8.6%) |  |
| Once a month | 799 (13.2%) | 4 (14.8%) | 1 (12.5%) | 0 (0.0%) | 804 (13.2%) |  |
| Every 2-3 months | 715 (11.8%) | 2 (7.4%) | 0 (0.0%) | 1 (14.3%) | 718 (11.8%) |  |
| Once or twice a year | 1,176 (19.4%) | 2 (7.4%) | 0 (0.0%) | 0 (0.0%) | 1,178 (19.3%) |  |
| Mean PGSI score (standard error) | 0.000 (0.000) | 1.148 (0.362) | 3.875 (1.126) | 14.143 (4.811) | 0.026 (0.528) | <0.001 |

^*^ PGSI = Problem Gambling Severity Index

^±^ NS-SEC = National Statistics Socio-economic classification

† An additional 1230 were removed from the sample for the sensitivity analysis due to missing data on gambling activity participation which meant it could not be determined if they only participated in lottery.

**Table S6** The results of the multinomial logistic regressions looking at the relationship between PGSI category and gambling frequency in a sample who do not solely gamble on lotteries. PGSI = Problem Gambling Severity Index.

|  | Model 1b | | |
| --- | --- | --- | --- |
|  | Relative risk ratio | 95% confidence interval | P-value |
| PGSI Category: No-Risk | Reference group | | |
| PGSI Category: Low-Risk |  |  |  |
| *Frequency of spending money on gambling activities in the past 12 months* |  |  |  |
| Ref: Once or twice a year | 1 | [1,1] | (.) |
| Every 2-3 months | 2.651 | [1.687,4.168] | (0.000) |
| Once a month | 4.211 | [2.709,6.546] | (0.000) |
| Less than once a week, more than once a month | 7.365 | [4.890,11.09] | (0.000) |
| Once a week | 5.715 | [3.849,8.483] | (0.000) |
| 2 or more times a week | 16.40 | [11.02,24.40] | (0.000) |
| *Age* |  |  |  |
| Ref: Age 18-34 | 1 | [1,1] | (.) |
| Age 35-49 | 0.462 | [0.368,0.578] | (0.000) |
| Age 50-64 | 0.300 | [0.231,0.391] | (0.000) |
| Age 65+ | 0.137 | [0.0922,0.204] | (0.000) |
| *Sex* |  |  |  |
| Ref: Male | 1 | [1,1] | (.) |
| Female | 0.301 | [0.241,0.377] | (0.000) |
| *NS-SEC*^±^  *(occupation)* |  |  |  |
| Ref: Higher managerial and professional occupations | 1 | [1,1] | (.) |
| Lower managerial and professional occupations | 1.197 | [0.846,1.694] | (0.311) |
| Intermediate occupations | 1.392 | [0.937,2.069] | (0.102) |
| Small employers and own account workers | 1.362 | [0.896,2.070] | (0.148) |
| Lower supervisory and technical occupations | 1.019 | [0.666,1.559] | (0.930) |
| Semi-routine occupations | 1.435 | [0.984,2.092] | (0.060) |
| Routine occupations | 1.136 | [0.771,1.674] | (0.518) |
| Never worked and long term unemployed | 1.619 | [0.598,4.382] | (0.343) |
| Other | 4.265 | [1.817,10.01] | (0.001) |
| *Frequency of alcohol intake in the past 12 months* |  |  |  |
| Ref: Almost every day | 1 | [1,1] | (.) |
| Five or six days a week | 1.189 | [0.676,2.091] | (0.549) |
| Three or four days a week | 1.348 | [0.886,2.051] | (0.163) |
| Once or twice a week | 1.003 | [0.673,1.494] | (0.990) |
| Once or twice a month | 0.956 | [0.619,1.477] | (0.840) |
| Once every couple of months | 1.163 | [0.720,1.881] | (0.537) |
| Once or twice a year | 1.294 | [0.780,2.146] | (0.318) |
| Not at all in the last 12 months/Non-drinker | 0.877 | [0.494,1.555] | (0.652) |
| *Long-term mental health disorder* |  |  |  |
| No | 1 | [1,1] | (.) |
| Yes | 1.420 | [1.017,1.983] | (0.039) |
| *Index of Multiple Deprivation for England and Scotland* |  |  |  |
| Least deprived- England | 1 | [1,1] | (.) |
| Least deprived- Scotland | 0.596 | [0.368,0.968] | (0.036) |
| 2- England | 1.040 | [0.730,1.481] | (0.829) |
| 2- Scotland | 0.668 | [0.432,1.031] | (0.069) |
| 3- England | 0.746 | [0.516,1.077] | (0.117) |
| 3- Scotland | 0.466 | [0.283,0.768] | (0.003) |
| 4- England | 0.926 | [0.648,1.323] | (0.673) |
| 4- Scotland | 0.659 | [0.419,1.037] | (0.071) |
| Most deprived- England | 0.932 | [0.650,1.335] | (0.700) |
| Most deprived- Scotland | 0.699 | [0.443,1.102] | (0.123) |
| PGSI Category: Moderate-Risk |  |  |  |
| *Frequency of spending money on gambling activities in the past 12 months* |  |  |  |
| Ref: Once or twice a year | 1 | [1,1] | (.) |
| Every 2-3 months | 2.505 | [1.063,5.904] | (0.036) |
| Once a month | 3.229 | [1.367,7.631] | (0.008) |
| Less than once a week, more than once a month | 4.646 | [2.065,10.45] | (0.000) |
| Once a week | 8.105 | [3.954,16.61] | (0.000) |
| 2 or more times a week | 37.68 | [18.67,76.03] | (0.000) |
| *Age* |  |  |  |
| Ref: Age 18-34 | 1 | [1,1] | (.) |
| Age 35-49 | 0.449 | [0.319,0.632] | (0.000) |
| Age 50-64 | 0.214 | [0.141,0.323] | (0.000) |
| Age 65+ | 0.0814 | [0.0424,0.156] | (0.000) |
| *Sex* |  |  |  |
| Ref: Male | 1 | [1,1] | (.) |
| Female | 0.300 | [0.208,0.432] | (0.000) |
| *NS-SEC (occupation)* |  |  |  |
| Ref: Higher managerial and professional occupations | 1 | [1,1] | (.) |
| Lower managerial and professional occupations | 1.445 | [0.788,2.648] | (0.234) |
| Intermediate occupations | 1.245 | [0.610,2.540] | (0.548) |
| Small employers and own account workers | 1.954 | [0.987,3.868] | (0.055) |
| Lower supervisory and technical occupations | 1.242 | [0.617,2.502] | (0.544) |
| Semi-routine occupations | 1.644 | [0.865,3.126] | (0.129) |
| Routine occupations | 1.471 | [0.780,2.772] | (0.233) |
| Never worked and long term unemployed | 4.25e-08 | NE | (0.996) |
| Other | 3.828 | [0.953,15.38] | (0.059) |
| *Frequency of alcohol intake in the past 12 months* |  |  |  |
| Ref: Almost every day | 1 | [1,1] | (.) |
| Five or six days a week | 0.950 | [0.427,2.112] | (0.900) |
| Three or four days a week | 0.850 | [0.479,1.509] | (0.579) |
| Once or twice a week | 0.780 | [0.464,1.314] | (0.351) |
| Once or twice a month | 0.526 | [0.284,0.973] | (0.040) |
| Once every couple of months | 0.636 | [0.313,1.290] | (0.210) |
| Once or twice a year | 0.764 | [0.366,1.595] | (0.473) |
| Not at all in the last 12 months/Non-drinker | 0.631 | [0.282,1.414] | (0.264) |
| *Long-term mental health disorder* |  |  |  |
| No | 1 | [1,1] | (.) |
| Yes | 1.592 | [0.972,2.607] | (0.065) |
| *Index of Multiple Deprivation for England and Scotland* |  |  |  |
| Ref: Least deprived- England | 1 | [1,1] | (.) |
| Least deprived- Scotland | 0.637 | [0.257,1.582] | (0.331) |
| 2- England | 1.038 | [0.529,2.036] | (0.913) |
| 2- Scotland | 0.661 | [0.293,1.492] | (0.319) |
| 3- England | 1.556 | [0.845,2.863] | (0.156) |
| 3- Scotland | 0.761 | [0.344,1.687] | (0.501) |
| 4- England | 1.252 | [0.665,2.354] | (0.486) |
| 4- Scotland | 0.698 | [0.307,1.588] | (0.391) |
| Most deprived- England | 2.309 | [1.281,4.162] | (0.005) |
| Most deprived- Scotland | 1.580 | [0.799,3.125] | (0.189) |
| PGSI Category: High-Risk |  |  |  |
| *Frequency of spending money on gambling activities in the past 12 months* |  |  |  |
| Ref: Once or twice a year | 1 | [1,1] | (.) |
| Every 2-3 months | 734843.5 | NE | (0.977) |
| Once a month | 3957435.7 | NE | (0.974) |
| Less than once a week, more than once a month | 1802449.1 | NE | (0.976) |
| Once a week | 5315742.6 | NE | (0.974) |
| 2 or more times a week | 34439866.3 | NE | (0.971) |
| *Age* |  |  |  |
| Ref: Age 18-34 | 1 | [1,1] | (.) |
| Age 35-49 | 0.501 | [0.298,0.844] | (0.009) |
| Age 50-64 | 0.257 | [0.140,0.470] | (0.000) |
| Age 65+ | 0.0678 | [0.0233,0.198] | (0.000) |
| *Sex* |  |  |  |
| Ref: Male | 1 | [1,1] | (.) |
| Female | 0.121 | [0.0616,0.237] | (0.000) |
| *NS-SEC (occupation)* |  |  |  |
| Ref: Higher managerial and professional occupations | 1 | [1,1] | (.) |
| Lower managerial and professional occupations | 4.275 | [0.977,18.70] | (0.054) |
| Intermediate occupations | 2.684 | [0.506,14.24] | (0.246) |
| Small employers and own account workers | 4.710 | [0.971,22.85] | (0.054) |
| Lower supervisory and technical occupations | 2.765 | [0.556,13.75] | (0.214) |
| Semi-routine occupations | 6.067 | [1.362,27.03] | (0.018) |
| Routine occupations | 6.417 | [1.474,27.94] | (0.013) |
| Never worked and long term unemployed | 5.365 | [0.579,49.68] | (0.139) |
| Other | 0.000000215 | NE | (0.998) |
| *Frequency of alcohol intake in the past 12 months* |  |  |  |
| Ref: Almost every day | 1 | [1,1] | (.) |
| Five or six days a week | 0.718 | [0.140,3.669] | (0.690) |
| Three or four days a week | 0.940 | [0.338,2.614] | (0.905) |
| Once or twice a week | 1.429 | [0.593,3.439] | (0.426) |
| Once or twice a month | 1.149 | [0.434,3.039] | (0.780) |
| Once every couple of months | 1.836 | [0.650,5.191] | (0.252) |
| Once or twice a year | 1.097 | [0.325,3.703] | (0.881) |
| Not at all in the last 12 months/Non-drinker | 2.029 | [0.657,6.270] | (0.219) |
| *Long-term mental health disorder* |  |  |  |
| No | 1 | [1,1] | (.) |
| Yes | 3.695 | [2.031,6.721] | (0.000) |
| *Index of Multiple Deprivation for England and Scotland* |  |  |  |
| Ref: Least deprived- England | 1 | [1,1] | (.) |
| Least deprived- Scotland | 0.794 | [0.141,4.483] | (0.794) |
| 2- England | 2.133 | [0.648,7.028] | (0.213) |
| 2- Scotland | 1.880 | [0.543,6.513] | (0.319) |
| 3- England | 1.891 | [0.589,6.068] | (0.284) |
| 3- Scotland | 1.289 | [0.347,4.789] | (0.704) |
| 4- England | 1.914 | [0.607,6.036] | (0.268) |
| 4- Scotland | 0.567 | [0.120,2.669] | (0.473) |
| Most deprived- England | 2.636 | [0.863,8.050] | (0.089) |
| Most deprived- Scotland | 1.999 | [0.602,6.643] | (0.258) |
| Observations | 9320 |  |  |
| Pseudo R^2^ | 0.186 |  |  |

^±^ NS-SEC = National Statistics Socio-economic classification

NE = 95% confidence interval calculation not feasible due to extremely small coefficient magnitude

**Table S7** The results of the zero-inflated negative binomial models looking at the relationship between PGSI score and gambling frequency in sample who do not solely gamble on lotteries. PGSI = Problem Gambling Severity Index.

|  | Model 2b | | |
| --- | --- | --- | --- |
|  | Incidence rate ratio | 95% confidence interval | P-value |
| **Count component** |  |  |  |
| *Frequency of spending money on gambling activities in the past 12 months* |  |  |  |
| Ref: Once or twice a year | 1 | [1,1] | (.) |
| Every 2-3 months | 0.926 | [0.445,1.929] | (0.838) |
| Once a month | 1.760 | [0.901,3.435] | (0.098) |
| Less than once a week, more than once a month | 1.113 | [0.587,2.111] | (0.744) |
| Once a week | 2.392 | [1.299,4.404] | (0.005) |
| 2 or more times a week | 3.946 | [2.177,7.152] | (0.000) |
| *Age* |  |  |  |
| Ref: Age 18-34 | 1 | [1,1] | (.) |
| Age 35-49 | 1.067 | [0.822,1.385] | (0.626) |
| Age 50-64 | 0.860 | [0.617,1.197] | (0.371) |
| Age 65+ | 0.569 | [0.336,0.962] | (0.035) |
| Sex |  |  |  |
| Ref: Male | 1 | [1,1] | (.) |
| Female | 0.567 | [0.424,0.759] | (0.000) |
| *NS-SEC^±^  (occupation)* |  |  |  |
| Higher managerial and professional occupations | 1 | [1,1] | (.) |
| Lower managerial and professional occupations | 1.422 | [0.878,2.302] | (0.152) |
| Intermediate occupations | 1.074 | [0.618,1.864] | (0.801) |
| Small employers and own account workers | 1.597 | [0.923,2.766] | (0.094) |
| Lower supervisory and technical occupations | 1.054 | [0.591,1.882] | (0.858) |
| Semi-routine occupations | 1.781 | [1.068,2.970] | (0.027) |
| Routine occupations | 1.910 | [1.115,3.269] | (0.018) |
| Never worked and long term unemployed | 1.379 | [0.418,4.550] | (0.598) |
| Other | 1.051 | [0.373,2.962] | (0.925) |
| *Frequency of alcohol intake in the past 12 months* |  |  |  |
| Ref: Almost every day | 1 | [1,1] | (.) |
| Five or six days a week | 1.474 | [0.749,2.899] | (0.261) |
| Three or four days a week | 1.178 | [0.726,1.912] | (0.507) |
| Once or twice a week | 1.408 | [0.905,2.190] | (0.129) |
| Once or twice a month | 1.430 | [0.884,2.315] | (0.145) |
| Once every couple of months | 1.701 | [0.974,2.972] | (0.062) |
| Once or twice a year | 1.815 | [0.992,3.322] | (0.053) |
| Not at all in the last 12 months/Non-drinker | 2.600 | [1.378,4.907] | (0.003) |
| *Long-term mental health disorder* |  |  |  |
| Ref: No | 1 | [1,1] | (.) |
| Yes | 1.533 | [1.052,2.232] | (0.026) |
| *Index of Multiple Deprivation for England and Scotland* |  |  |  |
| Ref: Least deprived- England | 1 | [1,1] | (.) |
| Least deprived- Scotland | 1.531 | [0.777,3.016] | (0.218) |
| 2- England | 1.396 | [0.859,2.269] | (0.178) |
| 2- Scotland | 1.291 | [0.740,2.253] | (0.368) |
| 3- England | 1.618 | [1.007,2.600] | (0.047) |
| 3- Scotland | 1.340 | [0.706,2.544] | (0.370) |
| 4- England | 1.152 | [0.719,1.846] | (0.557) |
| 4- Scotland | 0.933 | [0.516,1.689] | (0.819) |
| Most deprived- England | 1.630 | [1.017,2.614] | (0.042) |
| Most deprived- Scotland | 1.368 | [0.785,2.384] | (0.269) |
| **Zero component** |  |  |  |
| *Frequency of spending money on gambling activities in the past 12 months* |  |  |  |
| Ref: Once or twice a year | 1 | [1,1] | (.) |
| Every 2-3 months | 0.291 | [0.139,0.608] | (0.001) |
| Once a month | 0.240 | [0.127,0.453] | (0.000) |
| Less than once a week, more than once a month | 0.103 | [0.0540,0.198] | (0.000) |
| Once a week | 0.175 | [0.100,0.307] | (0.000) |
| 2 or more times a week | 0.0409 | [0.0228,0.0734] | (0.000) |
| *Age* |  |  |  |
| Ref: Age 18-34 | 1 | [1,1] | (.) |
| Age 35-49 | 2.862 | [2.060,3.976] | (0.000) |
| Age 50-64 | 4.976 | [3.369,7.349] | (0.000) |
| Age 65+ | 11.84 | [6.873,20.41] | (0.000) |
| *Sex* |  |  |  |
| Ref: Male | 1 | [1,1] | (.) |
| Female | 3.466 | [2.544,4.722] | (0.000) |
| *NS-SEC (occupation)* |  |  |  |
| Ref: Higher managerial and professional occupations | 1 | [1,1] | (.) |
| Lower managerial and professional occupations | 0.820 | [0.476,1.413] | (0.474) |
| Intermediate occupations | 0.666 | [0.350,1.267] | (0.215) |
| Small employers and own account workers | 0.654 | [0.341,1.256] | (0.203) |
| Lower supervisory and technical occupations | 0.814 | [0.407,1.628] | (0.561) |
| Semi-routine occupations | 0.774 | [0.434,1.382] | (0.387) |
| Routine occupations | 0.917 | [0.505,1.663] | (0.774) |
| Never worked and long term unemployed | 1.003 | [0.223,4.503] | (0.997) |
| Other | 0.114 | [0.00822,1.585] | (0.106) |
| *Frequency of alcohol intake in the past 12 months* |  |  |  |
| Ref: Almost every day | 1 | [1,1] | (.) |
| Five or six days a week | 1.102 | [0.478,2.543] | (0.819) |
| Three or four days a week | 0.905 | [0.479,1.709] | (0.757) |
| Once or twice a week | 1.281 | [0.713,2.300] | (0.407) |
| Once or twice a month | 1.692 | [0.905,3.163] | (0.099) |
| Once every couple of months | 1.344 | [0.673,2.685] | (0.402) |
| Once or twice a year | 1.348 | [0.662,2.742] | (0.410) |
| Not at all in the last 12 months/Non-drinker | 2.088 | [1.011,4.313] | (0.047) |
| *Long-term mental health disorder* |  |  |  |
| Ref: No | 1 | [1,1] | (.) |
| Yes | 0.660 | [0.426,1.023] | (0.063) |
| *Index of Multiple Deprivation for England and Scotland* |  |  |  |
| Ref: Least deprived- England | 1 | [1,1] | (.) |
| Least deprived- Scotland | 2.630 | [1.282,5.395] | (0.008) |
| 2- England | 1.023 | [0.558,1.875] | (0.941) |
| 2- Scotland | 1.801 | [0.924,3.511] | (0.084) |
| 3- England | 1.497 | [0.840,2.668] | (0.171) |
| 3- Scotland | 2.600 | [1.276,5.300] | (0.009) |
| 4- England | 0.980 | [0.532,1.808] | (0.950) |
| 4- Scotland | 1.678 | [0.812,3.470] | (0.162) |
| Most deprived- England | 0.988 | [0.550,1.776] | (0.969) |
| Most deprived- Scotland | 1.259 | [0.633,2.503] | (0.511) |
| Observations | 9320 |  |  |
| ln(α) | 2.479 | [1.935,3.178] | (0.000) |

*^±^* NS-SEC = National Statistics Socio-economic classification
